# Whole-Genome Sequencing and Comparative Analysis of *Mycobacterium brisbanense* Reveals a Possible Soil Origin and Capability in Fertiliser Synthesis

**DOI:** 10.1371/journal.pone.0152682

**Published:** 2016-03-31

**Authors:** Wei Yee Wee, Tze King Tan, Nicholas S. Jakubovics, Siew Woh Choo

**Affiliations:** 1 Genome Informatics Research Laboratory, High Impact Research Building (HIR) Building, University of Malaya, Kuala Lumpur, 50603, Malaysia; 2 Department of Oral Biology and Biomedical Sciences, Faculty of Dentistry, University of Malaya, Kuala Lumpur, 50603, Malaysia; 3 Center for Oral Health Research, School of Dental Sciences, Newcastle University, Framlington Place, Newcastle upon Tyne, United Kingdom; 4 Genome Solutions Sdn Bhd, Suite 8, Innovation Incubator UM, Level 5, Research Management & Innovation Complex, University of Malaya, 50603, Kuala Lumpur, Malaysia; University of Padova, Medical School, ITALY

## Abstract

*Mycobacterium brisbanense* is a member of *Mycobacterium fortuitum* third biovariant complex, which includes rapidly growing *Mycobacterium* spp. that normally inhabit soil, dust and water, and can sometimes cause respiratory tract infections in humans. We present the first whole-genome analysis of *M*. *brisbanense* UM_WWY which was isolated from a 70-year-old Malaysian patient. Molecular phylogenetic analyses confirmed the identification of this strain as *M*. *brisbanense* and showed that it has an unusually large genome compared with related mycobacteria. The large genome size of *M*. *brisbanense* UM_WWY (~7.7Mbp) is consistent with further findings that this strain has a highly variable genome structure that contains many putative horizontally transferred genomic islands and prophage. Comparative analysis showed that *M*. *brisbanense* UM_WWY is the only *Mycobacterium* species that possesses a complete set of genes encoding enzymes involved in the urea cycle, suggesting that this soil bacterium is able to synthesize urea for use as plant fertilizers. It is likely that *M*. *brisbanense* UM_WWY is adapted to live in soil as its primary habitat since the genome contains many genes associated with nitrogen metabolism. Nevertheless, a large number of predicted virulence genes were identified in *M*. *brisbanense* UM_WWY that are mostly shared with well-studied mycobacterial pathogens such as *Mycobacterium tuberculosis* and *Mycobacterium abscessus*. These findings are consistent with the role of *M*. *brisbanense* as an opportunistic pathogen of humans. The whole-genome study of UM_WWY has provided the basis for future work of *M*. *brisbanense*.

## Introduction

*Mycobacterium* is a genus within the Actinobacteria that includes well known human pathogens such as *Mycobacterium tuberculosis*, the causative agent of tuberculosis, and *Mycobacterium leprae*, the agent of leprosy. The nontuberculous mycobacteria (NTM), also known as environmental mycobacteria, are frequently isolated from environmental sources such as soil, water or animal materials [1,2]. Although these organisms tend to be found in the environment, many NTM are opportunistic pathogens, capable of causing human disease among immune-compromised individuals and even sometimes in immune-competent individuals [3].

The *M*. *fortuitum* complex includes numerous species, *M*. *fortuitum*, *Mycobacterium peregrinum*, *Mycobacterium mucogenicum*, *Mycobacterium mageritense*, *Mycobacterium farcinogenes*, *Mycobacterium septicum* [4] and an additional taxon with more than one species known as the third biovariant complex. This complex includes *Mycobacterium porcinum*, *Mycobacterium boenickei*, *Mycobacterium neworleansense*, *Mycobacterium houstonenense*, *Mycobacterium conceptionense* and *M*. *brisbanense* [4,5]. *M*. *brisbanense* is a rapidly growing *Mycobacterium* which has been isolated from human infections and from soil [6–8]. Species of the *M*. *fortuitum* complex can cause a variety of human infections including respiratory disease and wound infections [9,10]. In this paper, we described the genome of *M*. *brisbanense* UM_WWY (referred to as UM_WWY after this) isolated from the sputum of a 70-year-old Malaysian male patient with a diagnosis of latent tuberculosis. The genome of UM_WWY has been deposited at GenBank with the RefSeq ID of NZ_AUWS00000000.1.

## Results

### Genome Overview

**Fig 1:**
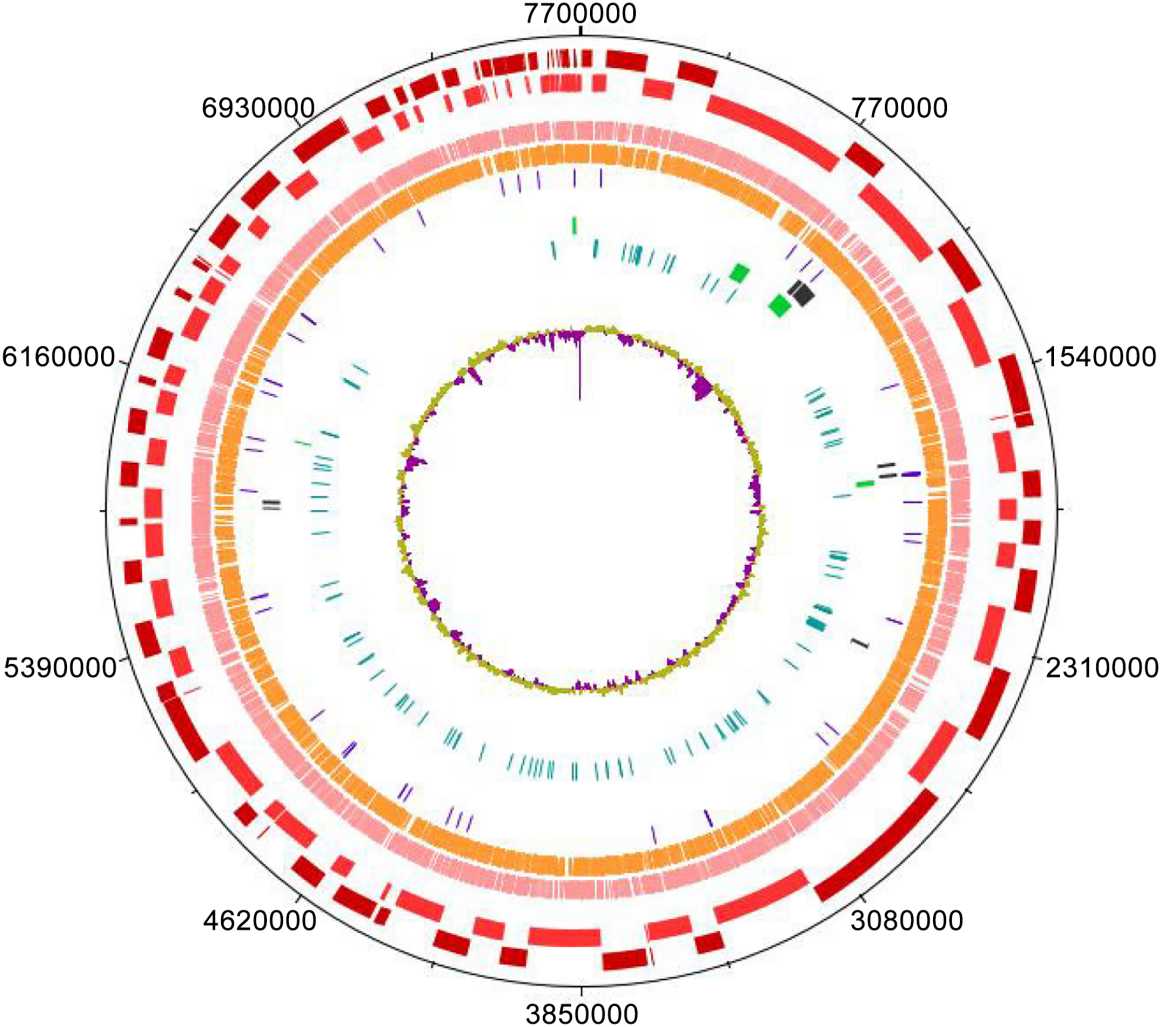
Schematic circular diagram of the UM_WWY genome. The following color bars represent: Dark red and light red are contigs, pink and orange represent forward and reverse CDS respectively, purple is tRNA, dark blue is genomic island, green is prophage, grey is virulence gene and the last track is the percentage of GC content.

The genome of UM_WWY was shot-gun sequenced using the Illumina HiSeq2000 platform, generating 63,311,594 raw sequencing reads. These reads were pre-processed to remove adaptor sequences and trimmed at a Phred quality score of 20 using CLC Genomic Workbench version 5.1 (CLC bio, Denmark). The assembly of 53,995,482 pre-processed reads resulted in 130 contigs. The assembly has a genomic size of 7,690,050bp with a G+C content of 66.4%. The sequenced genome of UM_WWY has an N75 value of 74,225bp, N50 value of 112,506bp and N25 size of 198,038bp, indicating the high quality of this assembly. The UM_WWY genome was BLAST-searched against NCBI database of known plasmids and no plasmid sequences were detected in the genome. An overview of the UM_WWY genome is shown in Fig 1. Interestingly, a 980kbp intact prophage was predicted in the sequenced genome of UM_WWY by the PHAST software. This large intact prophage observed within contig suggest it was recently inserted, contains 98 putative protein-coding genes and most of them have unknown functions (S1 Fig). Further studies on the functions of these hypothetical proteins may give better insights into the functions or impact of this prophage on the traits of UM_WWY. There was evidence that this prophage is currently active as we observed the presence of reads aligned to a closed form of the origin of insertion (*attP* site), indicating that lytic phage were likely to have been present in the cultures (S2 Fig).

### Phylogenetic Inference

**Fig 2:**
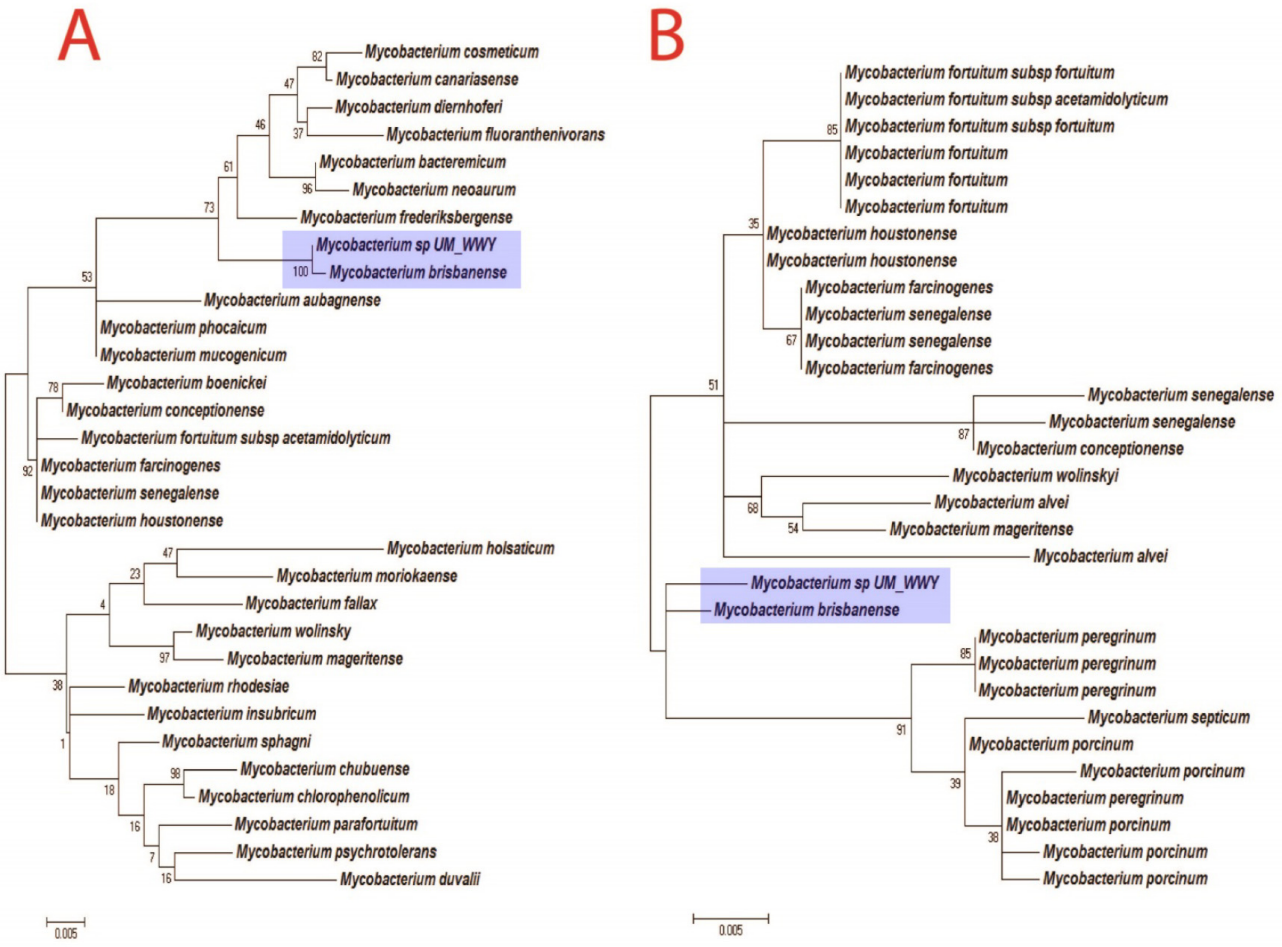
Phylogenetic inference. (A) *16S rRNA*-based phylogenetic tree. (B) *hsp65*-based phylogenetic tree. Maximum likelihood phylogenetic trees were inferred to identify the UM_WWY. Both results showed that *M*. *brisbanense* is the closest neighbour of UM_WWY.

The taxonomic position of UM_WWY was determined based on phylogenetic inferences of *16S rRNA* and *hsp65* marker genes. The *16S rRNA*-based phylogenetic tree was constructed using Hasegawa-Kishino-Yano DNA substitution model in MEGA5 [11]. The phylogenetic tree suggested that *M*. *brisbanense* is the closest neighbour of UM_WWY with a strong bootstrapping value of 100% (Fig 2A). Most of the closely related species of UM_WWY are members of the *M*. *fortuitum* complex, for example, *M*. *brisbanense*, *Mycobacterium cosmeticum*, *M*. *fortuitum*, *Mycobacterium housetonense*, *Mycobacterium senegalense*, *Mycobacterium boenickei* and *Mycobacterium mageritense*, suggesting that UM_WWY is likely a new member of *M*. *brisbanence*. Additionally, UM_WWY has the highest 16S rRNA gene sequence identity (99.8%) with *M*. *brisbanense* and second highest (98.53%) with *Mycobacterium cosmeticum*. The *hsp65*-based phylogenetic tree also showed that most of the closest neighbours of UM_WWY are also the members of *M*. *fortuitum* complex (Fig 2B). Besides that, it also supported that *M*. *brisbanense* as the closest neighbour of UM_WWY.

**Fig 3:**
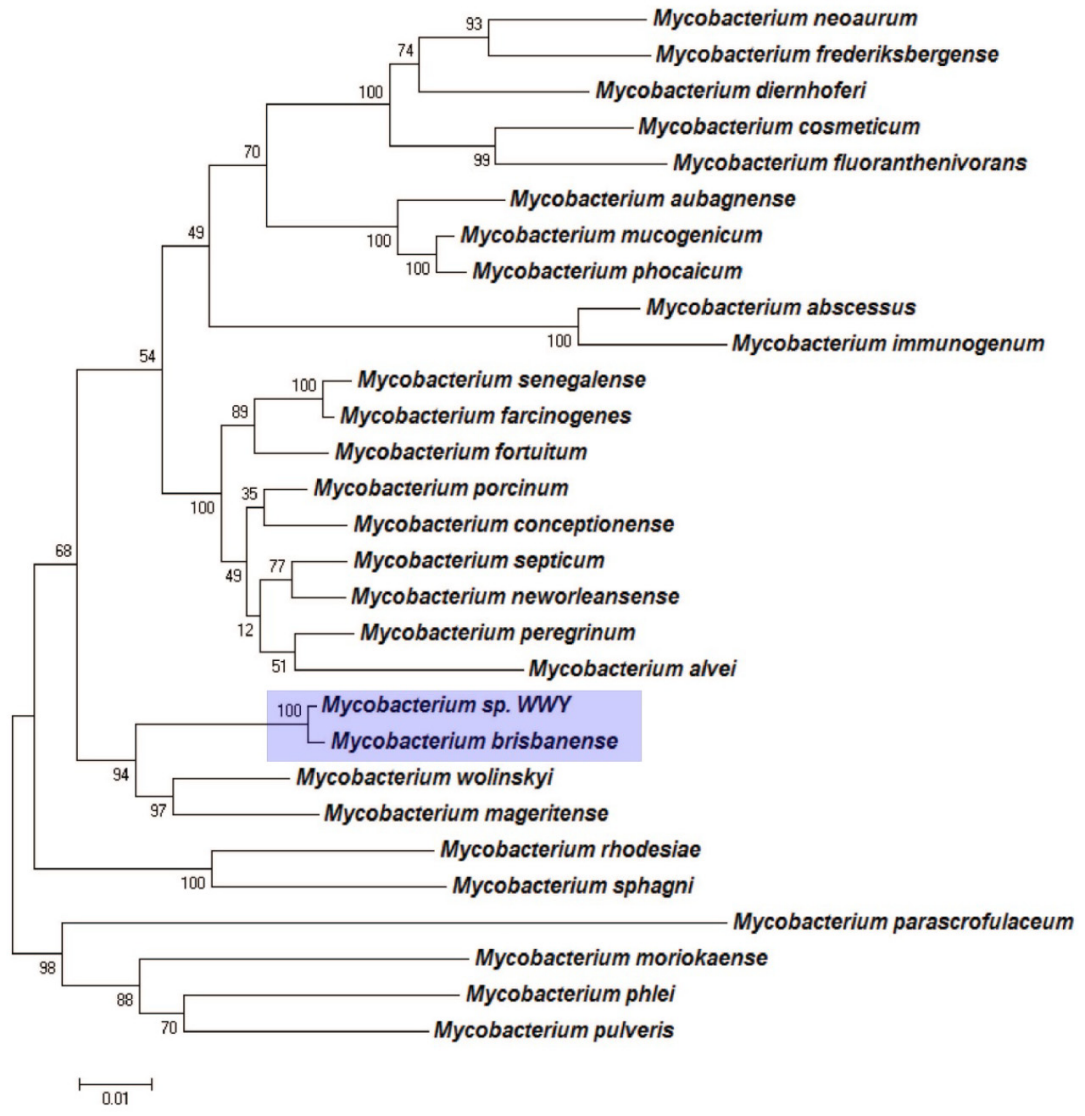
Supermatrix phylogenetic tree by concatenating *rpoB*, *tuf*, *sodA*, *16S rRNA* and *hsp65* gene sequences. Our results indicated that UM_WWY is closely related to *M*. *brisbanense* and other members in the *M*. *fortuitum* complex.

To further validate the taxonomic position of UM_WWY, we reconstructed a more robust tree using a multiple-genes approach based on the concatenated sequences of five housekeeping genes: *16S rRNA*, *rpoB*, *tuf*, *sodA* and *hsp65* genes [12]. The concatenated sequences were used to construct a phylogenetic tree using the same criteria as the above single gene-based approaches. Again, the multi-gene tree analysis showed results that were consistent with the single gene-based trees. *M*. *brisbanense* is the closest neighbour of UM_WWY with a sequence identity of 99% (Fig 3). Finally, a phylogenetic tree was built based on whole genome sequences of UM_WWY and its relatives (S1 Fig). Although genome sequences are not available for many species of the *M*. *fortuitum* complex, UM_WWY was found to cluster most closely to *M*. *fortuitum* complex species. Taken all together, our data support the view that UM_WWY is a *M*. *brisbanense* species and grouped together with the members of *M*. *fortuitum* complex.

### Functional Analysis

The sequenced genome of UM_WWY was annotated using the Rapid Subsystem Technology using Subsystem (RAST) pipeline [13]. We identified 7,504 protein-coding genes in the genome of UM_WWY. Of these, RAST successfully assigned functions to 2,362 (32%) genes (S2 Fig). Many genes were distributed in categories such as amino acid and derivatives (731), carbohydrates (644), cofactors, vitamins, prosthetic group pigments (413), fatty acids, lipids and isoprenoids (331) which are primarily responsible for the basic biological functions of the UM_WWY. Interestingly, we found 112 genes putatively involved in virulence, disease and defence. Of these, 59 genes were predicted to be involved in resistance to antibiotics and toxic compounds, whereas the others (53 genes) were putatively involved in host cell invasion and intracellular resistance. The antimicrobial resistance genes include arsenic resistance genes, cobalt-zinc-cadmium resistance genes, fluoroquinolones resistance genes and a beta-lactamase gene. The presence of these resistance genes indicates that UM_WWY may be resistant to certain heavy metals as well as fluoroquinolones and beta-lactam antibiotics (for example, penicillins, cephamycins and carbapenems) [14].

Three putative rRNAs and 73 tRNAs were identified in the genome of UM_WWY. The number of tRNA genes in UM_WWY is relatively high compared to other mycobacteria such as *Mycobacterium tuberculosis* (45) or *M*. *fortuitum* (55). Interestingly, further examination of these tRNA genes revealed that a large number of tRNA genes were clustered together in the genome of UM_WWY, forming a tRNA island (or a genomic region with a high density of tRNA genes) of approximately 3,175 bp, comprising 23 putative tRNA genesIt is likely that the tRNA island has been inserted into the UM_WWY genome through a horizontal gene transfer (HGT) event. The order of the tRNAs within this island is highly similar the order of tRNA genes in tRNA islands in four *Mycobacterium abscessus* strains isolated from the United States that we reported previously (S2 Table and S3 Fig) [15]. Although they are highly similar, there is one missing tRNA (tRNA-Leu) gene in the fourth position and one extra tRNA (tRNA-Arg) gene at the 5’ end of the tRNA island in the genome of UM_WWY. Therefore, it appears likely that these islands in UM_WWY and *Mycobacterium abscessus* have originated from the same source, probably from a transposition event since there is a transposase located immediately upstream of the tRNA island.

### Horizontally Transferred Genomic Islands

Our data showed that the genome size of UM_WWY strain is considerably larger than other *Mycobacterium* species that we analysed in this study. The genome of the clinically derived strain UM_WWY is approximately 1Mbp larger than *Mycobacterium marinum*, which has the second largest genome of the *Mycobacterium* species that were included here. The large genome size of UM_WWY may be due, at least in part, to HGT events involving the acquisition of foreign DNA over evolutionary time. Therefore, we were interested to search for possible horizontally transferred Genomic Islands (GIs) in the genome of UM_WWY. Genomic Islands are clusters of genes that are inserted into a bacterial genome during a single HGT event. These islands often play important roles in microbial evolution, virulence, drug resistance and/or adaptation to different environments [16].

Using IslandViewer [17,18], we identified 35 putative GIs (S1 Table), covering approximately 0.36Mbp of the UM_WWY genome. The large number of predicted GIs suggested that the HGT has played an important role in the evolution of UM_WWY and has contributed to the genome size. GI34 corresponds to transposon Tn554, a high frequency, site-specific transposable element that encodes three genes associated with transposition, *tnpA*, *tnpB* and *tnpC* [19]. Transposases were also identified in GI23 and GI33, indicating that these elements may also have inserted into the genome through transposition events. Previous studies have shown that GIs can play a role in the evolution of pathogenic *Mycobacterium* species [20,21]. Interestingly, we observed a putative virulence related gene, *espL in* GI35. The *espL* gene is one the members of *ESX-1* gene cluster for the type VII secretion systems that are responsible for the export of virulence factors [22]. However, the whole *ESX-1* cluster is required for full virulence in *Mycobacterium tuberculosis* [23]. Although we observed *espL* in GI35, other genes in the *ESX-1* cluster were not present indicating that the *espL* gene may not confer virulence traits on UM_WWY.

Additionally, we found a putative YefM toxin-antitoxin (TA) pathogenicity island (GI6) in the UM_WWY genome. TA systems have been reported to be associated with persistence and adaptability in *Mycobacterium tuberculosis* during infection [24]. Thus the insertion of this island might contribute to the virulence of UM_WWY.

### Comparative Genome Analysis

To retrieve more insights into the genome of UM_WWY, we compared UM_WWY with the *Mycobacterium* species that were found to be closely related according to our phylogenetic analyses above, namely *Mycobacterium mageritense* JR2009, *Mycobacterium septicum* DSM44393 and *M*. *fortuitum* DSM46621. Prior to the comparison of these genomes, we re-annotated all genomes using RAST server and the results were summarised in Table 1.

**Table 1:** Genome statistics of UM_WWY and three closely related species.

Strains	*Mycobacterium brisbanense* UM_WWY	*Mycobacterium septicum* DSM44393	*Mycobacterium mageritense* JR2009	*Mycobacterium fortuitum* DSM46621
**Genome Status**	Draft	Draft	Draft	Draft
**Genome Size (Mb)**	7.69	6.90	6.49	6.34
**Number of tRNAs**	73	52	72	55
**Number of rRNAs**	3	3	3	3
**Number of protein-coding genes**	7,504	6,598	6,238	6,077

**Fig 4:**
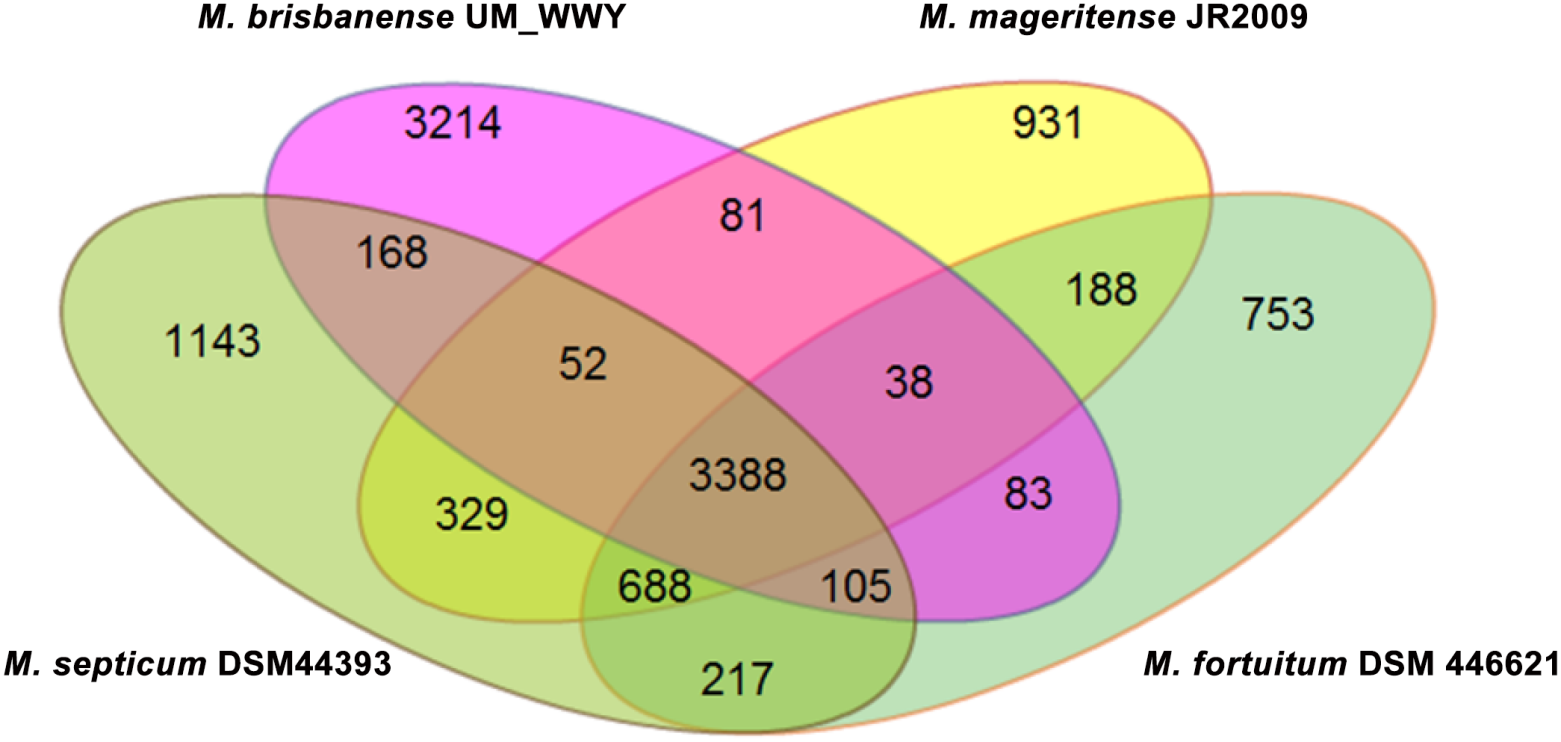
Venn diagram showing the gene distribution of *M*. *fortuitum* complex members and UM_WWY. The four genomes shared 3,388 gene clusters and UM_WWY contains the highest number of strain-specific genes.

UM_WWY has a genome size of approximately 7.69 Mbp, which is 0.79–1.35Mbp larger than the other three species. The difference was reflected in the high number of strain-specific gene clusters observed in the genome of UM_WWY (Fig 4). For instance, UM_WWY has the highest number of strain-specific genes (3,214 genes) compared to *Mycobacterium mageritense* JR2009 (931 genes), *Mycobacterium septicum* DSM44393 (1,143 genes) and *M*. *fortuitum* DSM46621 (753 genes). The high number of UM_WWY-specific genes suggests that this strain is very divergent from the most closely related species.

To further increase the stringency of the strain-specific genes, the genome of UM_WWY was further compared with genomes from 146 other mycobacterial strains. We clustered the genes of UM_WWY with the genes from the 146 strains composed of 27 different *Mycobacterium* species (S3 Table). Any UM_WWY-specific genes that overlapped the genes present in 146 mycobacterial strains were discarded. This stringent filtering step successfully reduced the set of UM_WWY-specific genes to 1,425. The presence of these 1,425 UM_WWY-specific genes may contribute to the unique traits or phenotypes of UM_WWY.

**Fig 5:**
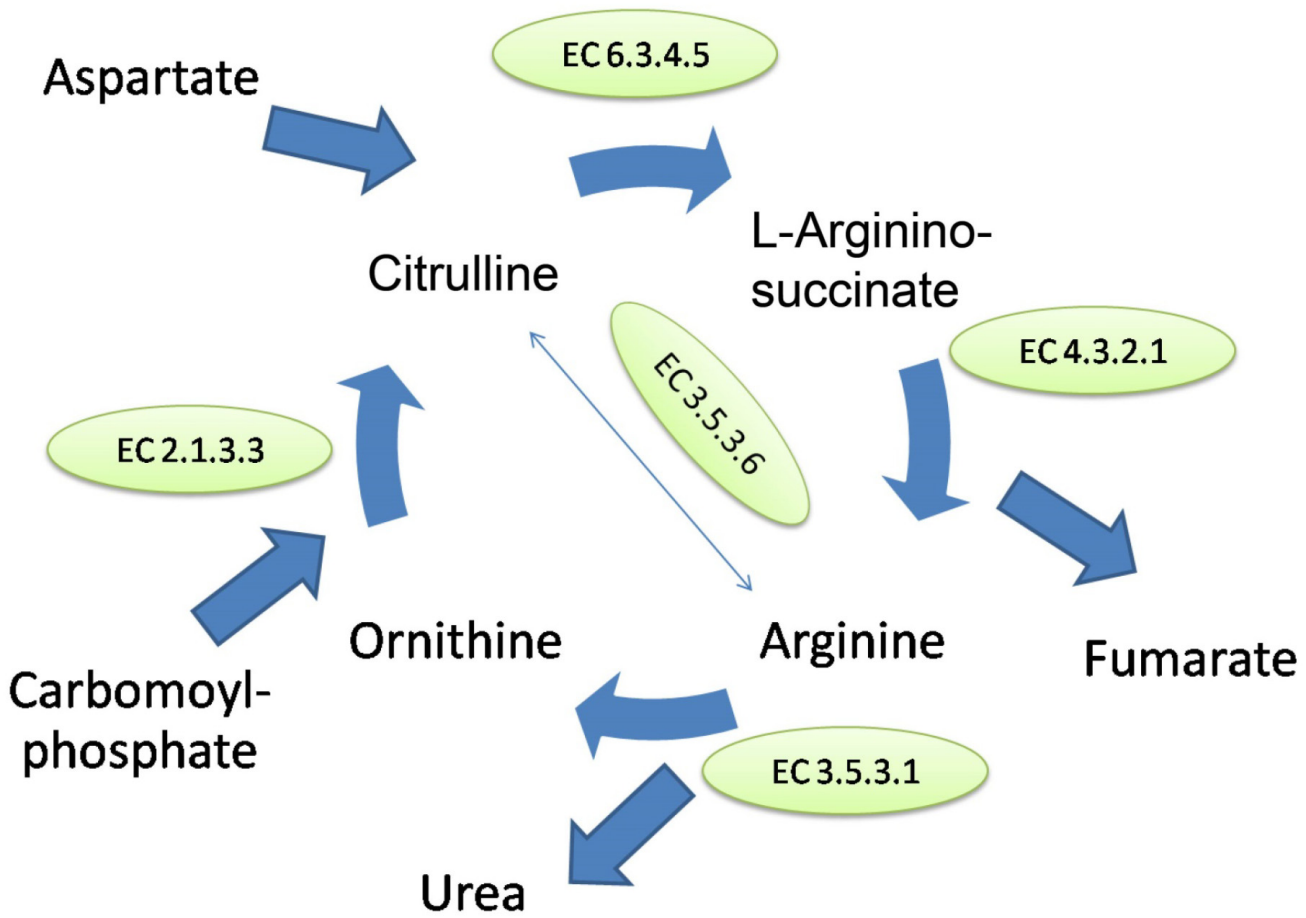
The pathway for urea cycle derived from KEGG pathway analysis. UM_WWY possess a gene encoding for a putative enzyme arginase (EC 3.5.3.1) which hydrolyzes arginine into ornithine and urea. EC 6.3.4.5 = Argininosuccinate synthetase; EC 4.3.2.1 = Argininosuccinate lyase; EC 2.1.3.3 = Ornithine transcarbamoylase.

Interestingly, further examinations of these genes revealed a gene encoding arginase (EC3.5.3.1), which is an enzyme found in some eubacteria and eukaryotes [25]. Arginase is one of the key enzymes in the urea cycle (Fig 5). This enzyme plays an important role in the hydrolysis of L-arginine to ornithine and urea. We examined the urea cycle KEGG (Kyoto Encyclopedia of Genes and Genomes) pathway and we found that the genome of UM_WWY also encodes other enzymes needed to form a complete urea cycle such as argininosuccinate synthetase (EC 6.3.4.5), argininosuccinate lyase (EC 4.3.2.1) and ornithine transcarbamoylase (EC 2.1.3.3) (Fig 5). In addition, gene encoding urease present in UM_WWY indicating that arginine or urea could be sources of nitrogen for this organism [26].Overall, this analysis indicates that UM_WWY is likely the first or the only known mycobacterial species which possesses the genetic basis for a complete urea cycle.

Apart from arginase, UM_WWY also encodes a putative sarcosine oxidase (*SOX*) operon which is absent in other mycobacterial species. *SOX* is an inducible enzyme in soil bacteria [27]. Lawrence and colleagues showed that SOX is a heterotetrameric enzyme and can catalyze the reaction of sarcosine to glycine, formaldehyde and H_2_O_2_.

$${\text{CH}}_{\text{3}}{\text{NHCH}}_{\text{2}}{\text{COOH + O}}_{\text{2}}{\text{+ H}}_{\text{2}}{\text{O -> HCHO + NH}}_{\text{2}}{\text{CH}}_{\text{2}}{\text{COOH + H}}_{\text{2}}{\text{O}}_{\text{2}}$$

The SOX operon is composed of five closely packed genes which encode the four SOX subunit (SOX α, SOX ß, SOX γ, SOX δ) and serine hydroxymethyltransferase (*gly*A). These genes are arranged in the order of *glyA*, followed by *SOX ß*, *SOX δ*, *SOX α* and *SOX γ* [28]. Surprisingly, a screen for these genes in the 146 mycobacterial genomes in MycoBase (manuscript under review), we found that UM_WWY is the only mycobacterial genome that possesses the entire *SOX* operon described by Lawrence and colleagues. The presence of the *SOX* operon may enable UM_WWY to grow on sarcosine as a sole source of carbon. Taken together, our data suggest that UM_WWY possesses many specific genes that are predicted to be related to the nitrogen metabolism, suggesting that this bacterium may play an important role in the urea cycle and nitrogen cycling compared to other mycobacterial species.

### Comparative Virulence Gene Analysis

We found 122 putative non-redundant virulence genes in the genome of UM_WWY. Almost all these virulence genes (121 genes) in the UM_WWY genome are shared by other human pathogens such as *Mycobacterium tuberculosis*. The genome of UM_WWY also contains genes such as *ahpC*, *katG*, *sodC* and *sodA* that encode enzymes involved in resistance to oxidative stress and might be important in combatting the oxidative burst during survival within macrophages [22]. Another virulence gene, *nuoG* encodes a protein that is one of the 14 subunits of type I NADH dehydrogenase and is a key virulence factor in *Mycobacterium tuberculosis* due to its ability to inhibit apoptosis of macrophages [29]. Thus, this gene allows the bacteria to escape from the natural cell death program by host cells.

Virulence genes were also identified in UM_WWY that encode proteins responsible for the synthesis of bacterial cell envelope, including *fbpA*, *fbpB*, *fbpC*, *erp*, *hbhA* and *mce*. The protein products of these genes are able to adhere and transport solutes across the mycobacterial cell wall in order to construct complex envelope lipids and proteins. For example, genes encoding Mce proteins, are organized in operons, and allow mycobacteria to enter into mammalian cell and survive inside the macrophage [30]. The UM_WWY genome has three *mce* operons, *mce1*, *mce3* and *mce4*. Moreover, UM_WWY also contains genes that encode proteins responsible for metal transporter. Metals such as iron, magnesium, copper and zinc are essential for the survival of bacteria in the environment or within the cell. However, excess levels of these metals are toxic to bacteria and thus metals transporters are required to maintain an appropriate level of metals within bacterial cells. The genes *irtA*, *irtB* and *ideR* in UM_WWY encode proteins that play a role in the acquisition of iron [22]. Another virulence gene, *mgtC*, encoding a protein responsible for transport of magnesium is also present in the genome of UM_WWY. Magnesium uptake is critical for intracellular pathogens to survive within macrophages as the magnesium level is very low [22].

There are many other putative virulence genes in UM_WWY such as four pairs of two component system genes (*devR-devS*; *mprA-mprB*; *phoP-phoR*; *prrA-prrB*), five sigma factors (*sigA*, *sigE*, *sigF*, *sigH*, *sigM*) that are involved in gene expression regulation, the *pknG* gene that acts as a protein kinase, and the metabolic genes (*mma4*, *mmpl8*, *pks2*, *papA1*, *papA2*, *stf0*) that are involved in lipid and fatty acid metabolism. The functions of other virulence-related genes remain unknown. The full list of the virulence genes in UM_WWY is shown in S4 Table.

After comparing with 30 other mycobacterial species (S4 Table), we found 25 virulence genes were highly conserved across all the Mycobacterium species. In comparison with *Mycobacterium tuberculosis*, UM_WWY has fewer virulence genes involved in the biosynthesis of phthiocerol dimycocerosate (PDIM) and phenolic glycolipid (PGL). PDIM is essential in prevention of acidification of phagosomes which disable enzymic digestion of internalized bacteria [31], while PGL is important in modulating the host immune response to infection [32]. For instance, of 20 genes in *Mycobacterium tuberculosis* that are involved in the synthesis of PDIM and PGL, UM_WWY has only three (*mas*, *fadD26* and *fadD28*). This suggests that the synthesis of PDIM and PGL might be disrupted in UM_WWY as previously shown in other mycobacterial species [33]. In addition, UM_WWY lacks of four genes encoding for phopholipase C-type enzymes which are involved in the virulence of *Mycobacterium tuberculosis* [34]. The absence of these genes suggests that UM_WWY is likely to be a less virulent pathogen than *Mycobacterium tuberculosis*.

## Discussion

In this paper, we report the first whole-genome sequence and analysis of *M*. *brisbanense* UM_WWY, which is a new member of *M*. *fortuitum* complex that was isolated from a 70-year-old Malaysian patient. Our molecular phylogenetic analyses using single marker gene and multiple gene approaches confirmed that UM_WWY is *M*. *brisbanense*.

One of the interesting observations in our analyses is that UM_WWY with a draft genome size of 7.69Mb (approximately 1MB bigger than the second largest *Mycobacterium* sp.) is the largest among the known genome size of the mycobacterial species that we used in this study. The large genome size of UM_WWY may reflect *M*. *brisbanense*’s diverse genomic structure probably driven by active HGT events. The evidence of large number of horizontally transferred GIs and prophages found in the genome of UM_WWY has further supported our view that the HGT has played important role in shaping the genome of UM_WWY. Recently it has been shown that a linear plasmid could be transferred *in vitro* from *Mycobacterium avium* to *Mycobacterium kansasii* and to *Mycobacterium bovis*, demonstrating that mycobacteria can transfer DNA between species naturally [35]. It is likely that *M*. *brisbanense* is particularly adept at the uptake and incorporation of genes from foreign sources. Interestingly, we also found evidence for the presence of an active bacteriophage in the *M*. *brisbanense* culture. In future studies, it would be interesting to characterise *M*. *brisbanense* bacteriophages in more detail since these may be important sources of HGT.

Strikingly, we found a large number of UM_WWY-specific genes compared to other mycobacteria, probably due to large genome of this bacterium. Further analysis of these specific genes has revealed that some of these genes are involved in pathway for urea synthesis. Interestingly, UM_WWY is the only *Mycobacterium* sp. (among all the mycobacterial genomes that we analysed in this study) that has the complete set of genes involved in the urea cycle including an arginase that can potentially convert arginine to ornithine and urea. It has been reported that arginine can be used by macrophages to produce reactive nitrogen intermediates such as nitric oxide that serve as antimicrobial agents against invading pathogens [36]. It has also been shown in *Helicobacter pylori* that the presence of arginase is able to hydrolyze arginine and inhibit the production of these reactive nitrogen intermediates, allowing the survival of the bacteria in the host by inhibiting the host from producing nitric oxide [37]. Thus, it is possible that the arginase gene may play an important role in the virulence of UM_WWY, for example, by inhibiting the production of reactive nitrogen intermediates.

According to a previous report from Amon and colleagues, *Mycobacterium smegmatis* contains the highest number of predicted nitrogen metabolism genes among the sequenced mycobacteria [38]. This is probably due to the fact that the *Mycobacterium smegmatis* naturally lives in the soil. Interestingly, we found that UM_WWY also has all of these genes that are involved in the nitrogen metabolism, suggesting that UM_WWY is also likely to exist primarily in the soil. Although UM_WWY was isolated from a clinical infection, there have been reports of isolation of *M*. *brisbanense* from soil. The high number of putative virulence genes found in the genome of UM_WWY might enable this bacterium to transition between its primary environmental habitats to growth in humans as an opportunistic pathogen. If HGT has played an important role in shaping the genome of UM_WWY, as suggested based on our analyses such as the presence of GIs, it is possible that this bacterium may continue acquiring new genes and may become increasingly more virulent in the future.

In this study, we revealed a region with a high density of tRNA genes (also predicted as a GI) in the genome of UM_WWY that is highly similar to the uncommon tRNAs islands found in some mycobacterial strains such as the highly antibiotic resistant strain *Mycobacterium bollettii* M24 and some *Mycobacterium massiliense* strains from the United States [15]. Although this is an interesting observation, the biological significance of this tRNA island is not clear. It is possible that the large tRNA gene cluster may be associated with elevated levels of protein synthesis, or possibly tied in with pathogenicity. Further experimental works such as cell invasion assays and genetic manipulation may help to give better insights into the pathogenic potential of *M*. *brisbanense* and the roles of specific genetic factors.

## Conclusion

Here we present the first genome sequence of *M*. *brisbanense*. Comparative analyses suggest that UM_WWY is likely to be primarily a soil bacterium and may have industrial potential for the synthesis of urea for use in fertilizers. The presence of large numbers of virulence-associated genes and the apparent capacity for HGT may allow UM_WWY to evolve into a more serious human pathogen. This whole-genome sequence will provide a model genome for studying *M*. *brisbanense* in the future.

## Methodology

### Library Construction and Whole-Genome Sequencing

UM_WWY was isolated from the sputum of a 70-year-old male patient with a diagnosis of latent tuberculosis in Malaysia. The DNA of UM_WWF was obtained from Professor Dr. Ngeow Yun Fong from the Faculty of Medicine, University of Malaya. The DNA sample was fragmented using Covaris S2 for 120 seconds at temperature of 5.5–6.0 degree Celsius. The quantity and quality of fragmented materials were examined using the Agilent BioAnalyzer 2100. The sample was size selected using Invitrogen 2% agarose E-gels. Fragments with adapter molecules at both ends further underwent 10 cycles of PCR for library construction. The Agilent BioAnalyzer 2100 was used to validate the constructed genomic library and the pool of 8pM was loaded onto Illumina HiSeq2000 flow cell v3 for 100bp paired-end sequencing at approximately 1,000X coverage.

### Raw Data Preprocessing and Genome Assembly

Exact duplicates and reverse complement duplicate reads were filtered using standalone PRINSEQ lite version 0.20 [39]. The final reads pre-processing based on Phred quality score of 20 and the genome assembly were performed using CLC Genomic Workbench version 5.1. The assembly of the reads was carried out at length faction of 0.7 and similarity fraction of 0.9, accepting minimal size of contig of 500bp.

### Phylogenetic Inferences

The candidates used in the classification of UM_WWY were identified using the updated version of Bioinformatics Bacteria Identification Tool—leBIBI V5 [40]. The 16S rRNA-based phylogenetic tree was constructed using Hasegawa-Kishino-Yano DNA substitution model in Molecular Evolutionary Genetics Analysis (MEGA5) [11] tool with bootstrap value of 500. Using the same criteria as the *16S rRNA* phylogenetic study, *hsp65*-based maximum likelihood phylogenetic was inferred using Tamura-Nei DNA substitution model. Five selected bacterial classification markers, *16S*, *hsp65*, *rpoB*, *tuf*, *sodA* and *rRNA* from all the possible closest species were extracted and the complete gene sequences were concatenated for phylogenetic tree construction.

### Genome Annotation

The sequenced genome of UM_WWF was submitted to the RAST server for annotation [13]. Genomic islands in UM_WWY were predicted by IslandViewer which integrates several different approaches to predict the GIs in genomes [18] These approaches include the sequence composition based SIGI-HMM [41] and IslandPath-DIMOB [42,43] that have been shown to have specificity of 86% to 92% and accuracy of 86%, and the comparative genomics approach IslandPick [18,42,44]. The generated results from IslandViewer were further filtered by eliminating the islands situated within two contigs [44]. To predict virulence genes, the RAST-predicted protein sequences were BLAST searched against Virulence Factors Database (VFDB) [45–47] and results were filtered using an in-house Perl scripts to select orthologous genes that are at least 50% sequence identity and 50% sequence completeness.

### Comparative Analysis

The predicted protein sequences of UM_WWY, *Mycobacterium mageritense* JR2009, *Mycobacterium septicum* DSM44393 and *M*. *fortuitum* DSM4662 were clustered into orthologs. In order to cluster orthologs into the same group, protein sequences must be at least 50% identity and 50% coverage to each other. To avoid false prediction, the orthologs that are not present in all the strains were further BLAST-searched against the whole-genome sequences to check the existence of the gene.

The same methods were used to identify the species-specific genes in UM_WWY with the changing of reference sequences from three *M*. *fortuitum* complex strains to 146 Mycobacterium species strains covering 27 different mycobacterial species.

## Supporting Information

S1 Fig Structure of a predicted intact prophage. Different colors of bar indicated different categories of genes in the prophage of UM_WWY.
(TIF)

S2 Fig Lytic phage screening. The predicted intact prophage sequence was extracted from the genome of UM_WWY. Both ends (broken Attachment site (AttP)) of the prophage were manually joined together into circular form (mimicking a lytic phage). All raw sequencing reads were mapped to the joint point of the broken Attachment site. We found 927 paired-end reads covered the joint point, indicating the prophage was likely excised.
(TIF)

S3 Fig Functional classification of the predicted protein-coding genes in the genome of UM_WWY. (TIF)

S4 Fig The order of tRNA genes in the tRNA islands found in the genome of UM_WWY and four *Mycobacterium abscessus* strains. The order of tRNA genes are highly similar among the five strains except one tRNA gene in the 4^th^ position was missing and one extra tRNA found in the UM_WWY genome.
(TIF)

S1 File Phylogenetic tree of core genome SNP. (PDF)

S1 Table Predicted GIs in the genome of UM_WWY. Selected genes of GIs are shown in this table.
(DOCX)

S2 Table Composition of the tRNAs island for UM_WWY and four *M*. *abscessus* strains. (DOCX)

S3 Table List of *Mycobacterium* genomes used in the clustering of the UM_WWY genes. (DOCX)

S4 Table Predicted virulence genes across the UM_WWY and 30 other ycobacterial genomes. “/” means presence of the predicted virulence gene. “-” means absence of the predicted virulence gene.
(DOCX)
