# Supplementary figures and images for: Whole-Genome Sequencing and Comparative Analysis of Mycobacterium brisbanense Reveals a Possible Soil Origin and Capability in Fertiliser Synthesis
Source: PLoS One. 2016 Mar 31;11(3):e0152682. doi: 10.1371/journal.pone.0152682 (PMC4816395; doi:10.1371/journal.pone.0152682)

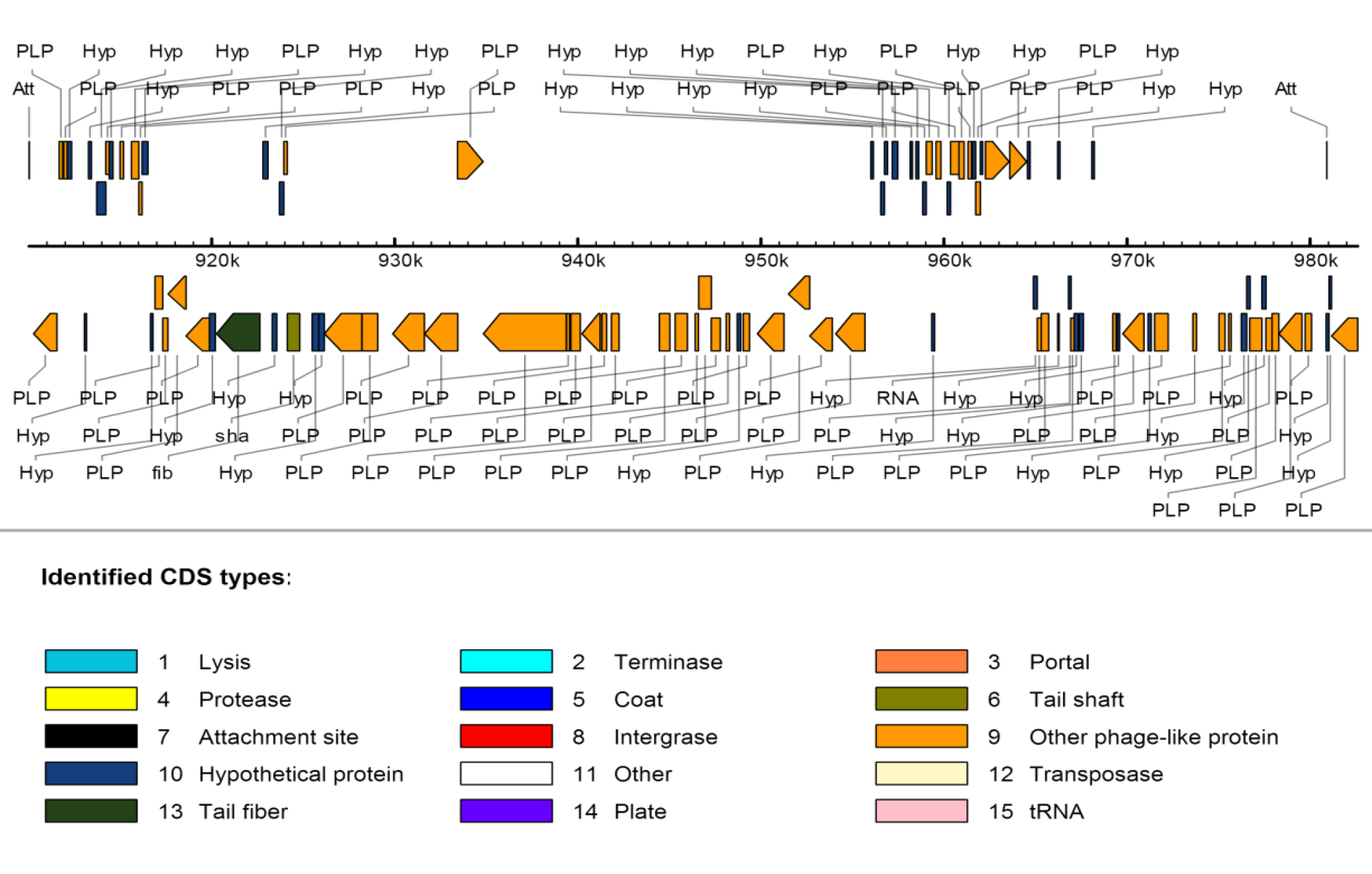

Supplement: S1 Fig — Different colors of bar indicated different categories of genes in the prophage of UM_WWY. (TIF) [file pone.0152682.s001.tif]

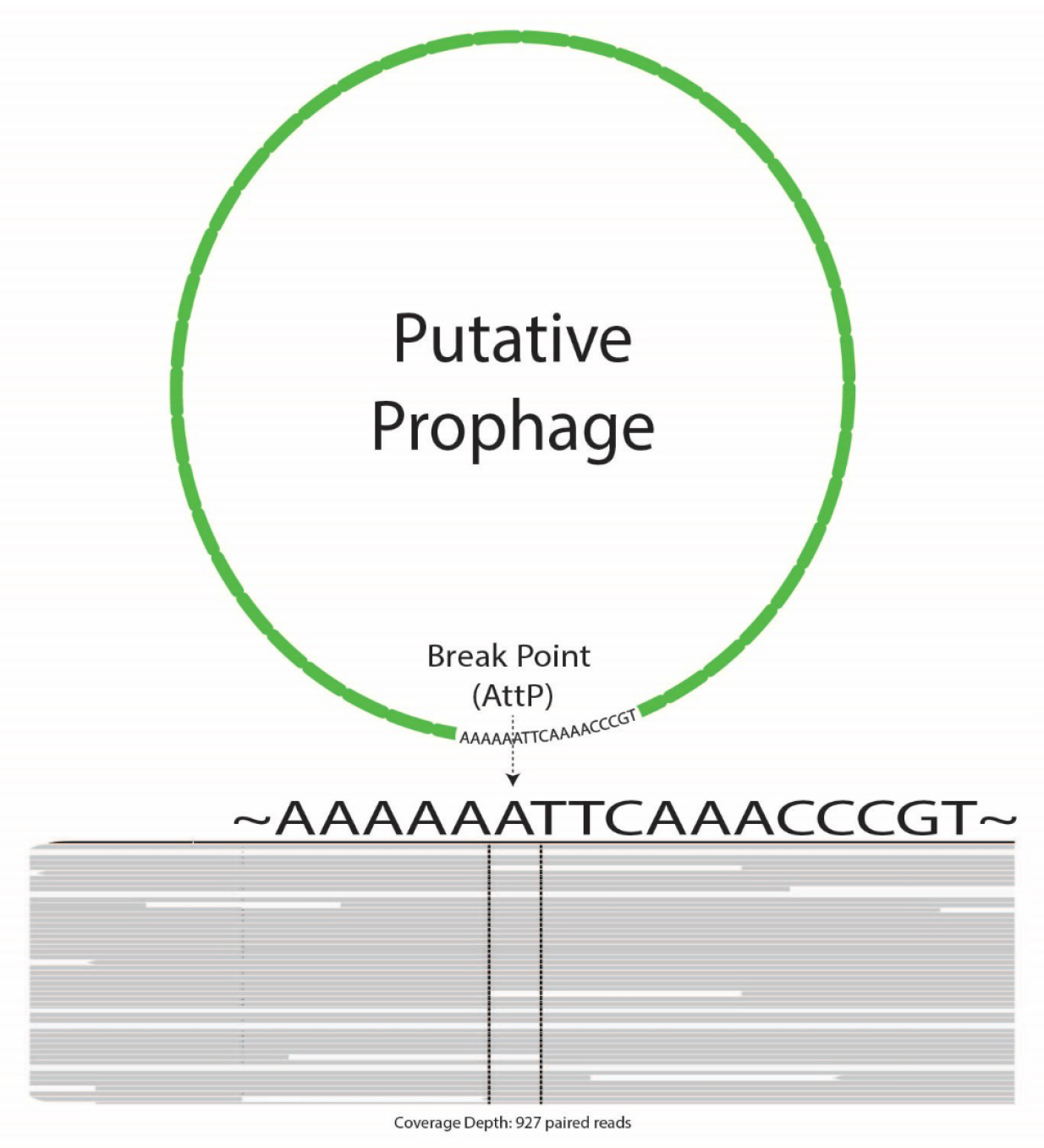

Supplement: S2 Fig — The predicted intact prophage sequence was extracted from the genome of UM_WWY. Both ends (broken Attachment site (AttP)) of the prophage were manually joined together into circular form (mimicking a lytic phage). All raw sequencing reads were mapped to the joint point of the broken Attachment site. We found 927 paired-end reads covered the joint point, indicating the prophage was likely excised. (TIF) [file pone.0152682.s002.tif]

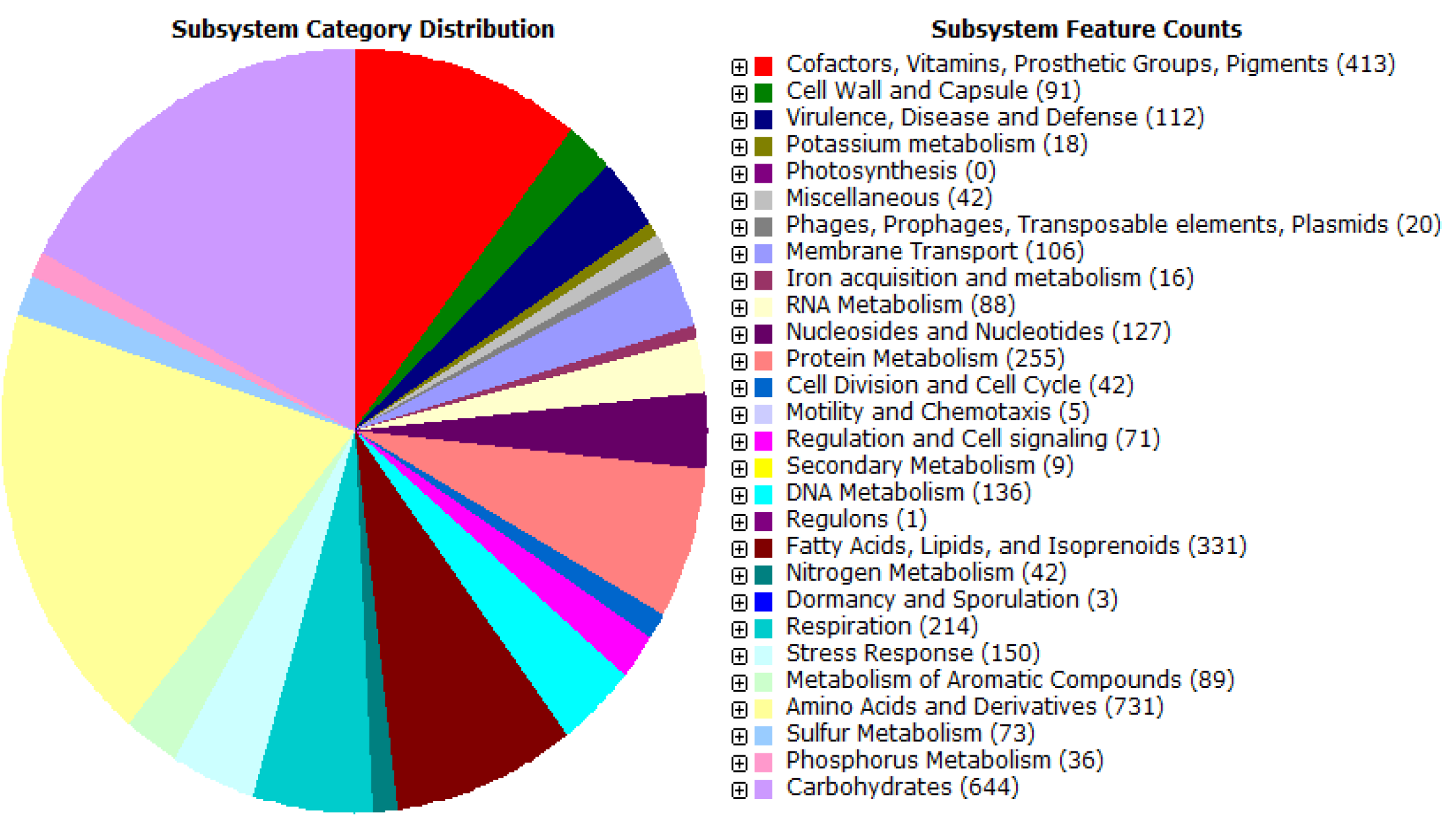

Supplement: S3 Fig — (TIF) [file pone.0152682.s003.tif]

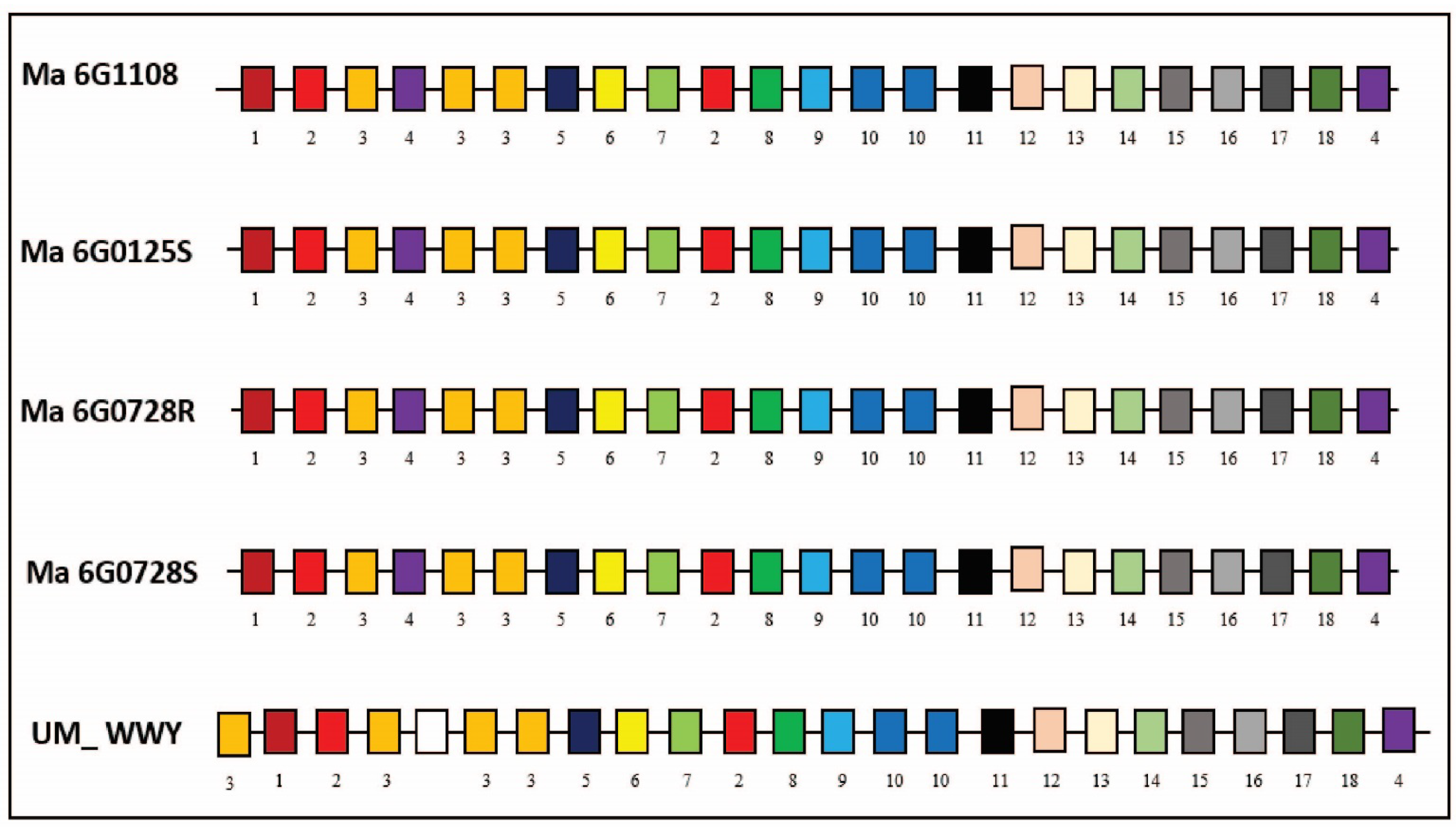

Supplement: S4 Fig — The order of tRNA genes are highly similar among the five strains except one tRNA gene in the 4th position was missing and one extra tRNA found in the UM_WWY genome. (TIF) [file pone.0152682.s004.tif]
