# Supplementary material for: Whole-Genome Sequencing and Comparative Analysis of Mycobacterium brisbanense Reveals a Possible Soil Origin and Capability in Fertiliser Synthesis
Source: PLoS One. 2016 Mar 31;11(3):e0152682. doi: 10.1371/journal.pone.0152682 (PMC4816395; doi:10.1371/journal.pone.0152682)
Supplement: S1 File — (PDF) [file pone.0152682.s005.pdf]

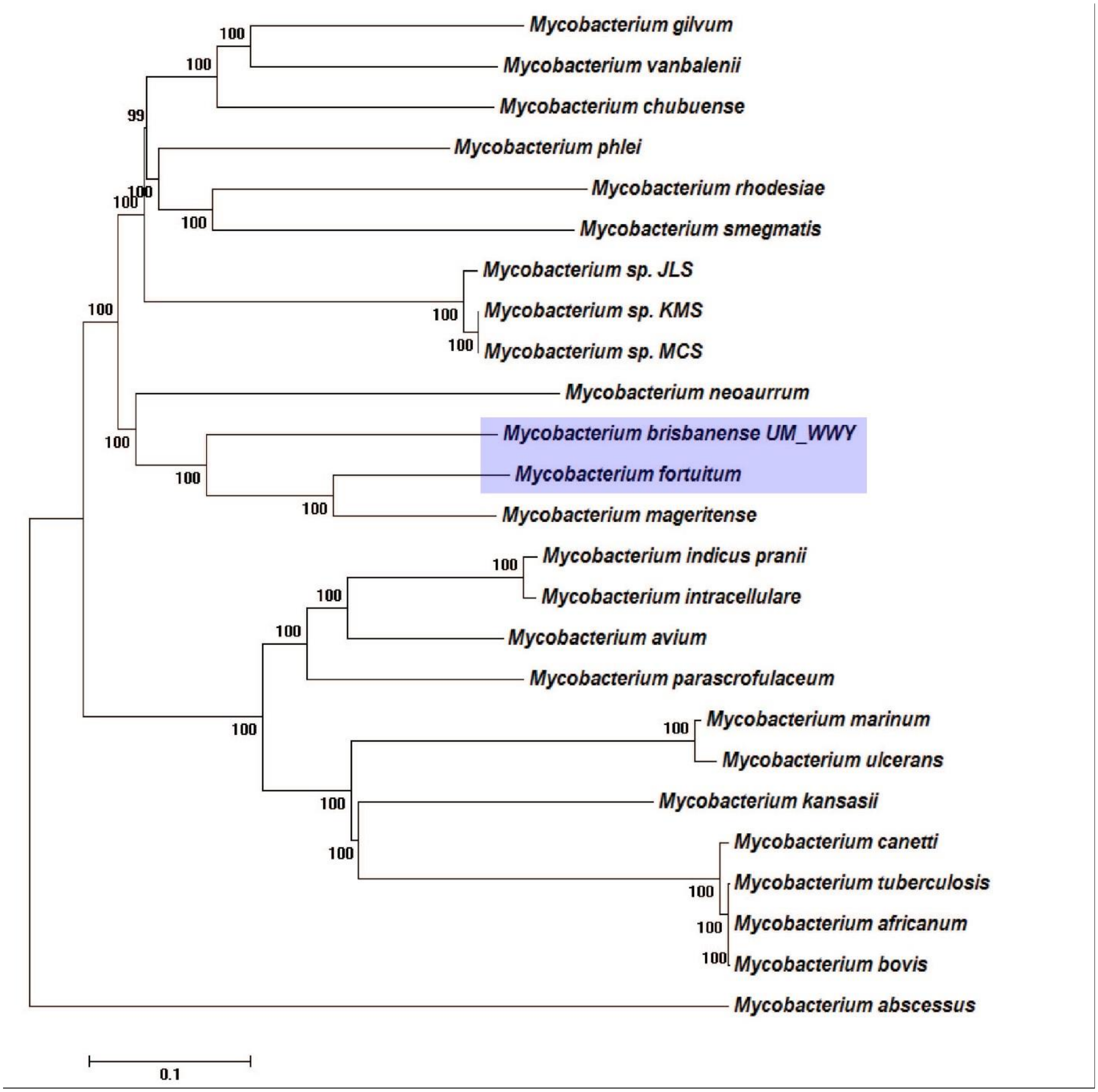

S1 File: Phylogenetic tree of core genome SNP.

As anticipated, the tree is in concordance with the major known relationships for *Mycobacterium* spp. (Tortoli, 2012). Interestingly, the generated tree has also clearly separated the rapid-growing mycobacteria (RGM) and slow-growing mycobacteria (SGM). For instance, the known slow growers, the *Mycobacterium tuberculosis* complex (*Mycobacterium tuberculosis*, *Mycobacterium africanum* and *Mycobacterium bovis*), *Mycobacterium ulcerans*, *Mycobacterium marinum* and the *Mycobacterium avium* complex species (*Mycobacterium avium*, *Mycobacterium intracellulare*) are clustered together. For the known rapid-growers such as *Mycobacterium vanbaalenii*, *Mycobacterium gilvum*, *Mycobacterium magmatism*,

Mycobacterium fortuitum complex members (*Mycobacterium fortuitum*, *Mycobacterium mageritense*, and *Mycobacterium brisbananse*) are clustered into a large clade and clearly separated from other rapid growing mycobacteria, as well as the free-living mycobacterial strains such as *Mycobacterium sp.* JLS, *Mycobacterium sp.* KMS and *Mycobacterium sp.* MCS. The result is also in concordance with the genus tree from previous study which also generated the phylogenetic tree based on concatenation of the core genes (Librado *et al.*, 2014). Although we are unable to indicate that UM_WWY is the *Mycobacterium brisbananse* from this tree as there is currently no *Mycobacterium brisbananse* whole genome is available as reference, but UM_WWY again is reside inside the *Mycobacterium fortuitum* complex.

Tortoli, E. (2012). Phylogeny of the genus Mycobacterium: many doubts, few certainties. *Infect Genet Evol*, *12*(4), 827-831. doi: 10.1016/j.meegid.2011.05.025

Librado, P., Vieira, F. G., Sanchez-Gracia, A., Kolokotronis, S. O., & Rozas, J. (2014). Mycobacterial phylogenomics: an enhanced method for gene turnover analysis reveals uneven levels of gene gain and loss among species and gene families. *Genome Biol Evol*, *6*(6), 1454-1465. doi: 10.1093/gbe/evu117
