# Supplementary material for: Whole-Genome Sequencing and Comparative Analysis of Mycobacterium brisbanense Reveals a Possible Soil Origin and Capability in Fertiliser Synthesis
Source: PLoS One. 2016 Mar 31;11(3):e0152682. doi: 10.1371/journal.pone.0152682 (PMC4816395; doi:10.1371/journal.pone.0152682)
Supplement: S1 Table — Selected genes of GIs are shown in this table. (DOCX) [file pone.0152682.s006.docx]

**S1 Table: Predicted GIs in the genome of UM_WWY.** Selected genes of GIs are shown in this table.

| **GI** | **Length (bp)** | **GC content (%)** | **Key Genes** |
| --- | --- | --- | --- |
| GI1 | 4,904 | 61.6 | - Hypothetical proteins |
| GI2 | 4,888 | 62.6 | - Undecaprenyl-diphosphatase (EC 3.6.1.27) |
| GI3 | 8,220 | 58.4 | - Phage like proteins |
| GI4 | 20,833 | 58.8 | - Phage like proteins  - Phage tai proteins |
| GI5 | 60,965 | 57.9 | - Phage portal  - Phage terminase |
| GI6 | 8,236 | 65.0 | - Toxin and antitoxin proteins |
| GI7 | 4,820 | 64.0 | - Hypothetical proteins |
| GI8 | 7,667 | 62.9 | - Hypothetical proteins |
| GI9 | 16,572 | 63.5 | - tRNAs gene cluster |
| GI10 | 17,458 | 63.2 | - Mobile element proteins |
| GI11 | 5,854 | 62.9 | - Proline iminopeptidase  - Esterase |
| GI12 | 4,158 | 65.7 | - Hypothetical proteins |
| GI13 | 4,170 | 63.3 | - Hypothetical proteins |
| GI14 | 7,157 | 63.0 | - Hypothetical proteins |
| GI15 | 5,634 | 61.8 | - Hypothetical proteins |
| GI16 | 7,625 | 62.5 | - Multiple enzymes |
| GI17 | 11,105 | 62.5 | - RD1 region associated protein Rv3879c |
| GI18 | 4,152 | 61.4 | - Ribose ABC transport system |
| GI19 | 4,641 | 58.2 | - Hypothetical proteins |
| GI20 | 7,002 | 59.4 | - Hypothetical proteins |
| GI21 | 4,208 | 61.0 | - Cobyrinic acid a,c-diamide synthase |
| GI22 | 5,290 | 59.8 | - Hypothetical proteins |
| GI23 | 12,813 | 63.8 | - Putative transposase, IS891/IS1136/IS1341 |
| GI24 | 21,952 | 61.6 | - Pyruvate dehydrogenase E1  - Long-chain-fatty-acid--CoA ligase  - 3-oxoacyl-[acyl-carrier-protein] reductase( EC:1.1.1.100 ) |
| GI25 | 6,476 | 62.3 | - RD1 region associated protein Rv3879c |
| GI26 | 6,368 | 64.0 | - Exonuclease  - Hypothetical proteins |
| GI27 | 13,800 | 64.4 | - Replicative DNA helicase (EC 3.6.1.-) |
| GI28 | 10,268 | 63.3 | - Hypothetical proteins |
| GI29 | 12,226 | 62.6 | - Ribose ABC transport system |
| GI30 | 14,815 | 61.0 | - Mobile element proteins |
| GI31 | 4,210 | 61.0 | - Hypothetical proteins |
| GI32 | 11,454 | 57.8 | - Hypothetical proteins |
| GI33 | 9,150 | 65.0 | - Putative transposase |
| GI34 | 10,961 | 65.5 | - Tn554 transposon |
| GI35 | 7,176 | 63.6 | - Hypothetical proteins |
