# Supplementary material for: Whole-Genome Sequencing and Comparative Analysis of Mycobacterium brisbanense Reveals a Possible Soil Origin and Capability in Fertiliser Synthesis
Source: PLoS One. 2016 Mar 31;11(3):e0152682. doi: 10.1371/journal.pone.0152682 (PMC4816395; doi:10.1371/journal.pone.0152682)
Supplement: S2 Table — (DOCX) [file pone.0152682.s007.docx]

**S2 Table: Composition of the tRNAs island for UM_WWY and four *M*. *abscessus* strains.**

| **Strains** | **tRNAs** | | | | | | | | | | | | | | | | | |
| --- | --- | --- | --- | --- | --- | --- | --- | --- | --- | --- | --- | --- | --- | --- | --- | --- | --- | --- |
|  | **Arg** | **Asn** | **Asp** | **Cys** | **Gln** | **Glu** | **Gly** | **His** | **Ile** | **Leu** | **Lys** | **Met** | **Phe** | **Pseudo** | **Ser** | **Thr** | **Trp** | **Val** |
| ***M. brisbanense* UM_WWY** | 4 | 1 | 1 | 1 | 2 | 1 | 1 | 1 | 2 | 1 | 1 | 1 | 1 | 1 | 1 | 1 | 1 | 1 |
| ***M. abscessus* strains** | 3 | 1 | 1 | 1 | 2 | 1 | 1 | 1 | 2 | 2 | 1 | 1 | 1 | 1 | 1 | 1 | 1 | 1 |
