# Supplementary material for: Whole-Genome Sequencing and Comparative Analysis of Mycobacterium brisbanense Reveals a Possible Soil Origin and Capability in Fertiliser Synthesis
Source: PLoS One. 2016 Mar 31;11(3):e0152682. doi: 10.1371/journal.pone.0152682 (PMC4816395; doi:10.1371/journal.pone.0152682)
Supplement: S3 Table — (DOCX) [file pone.0152682.s008.docx]

**S3 Table: List of *Mycobacterium* genomes used in the clustering of the UM_WWY genes.**

| **Species** | **Strain name** |
| --- | --- |
| *Mycobacterium abscessus* | 4S0116S |
| *Mycobacterium abscessus* | 3A0119R |
| *Mycobacterium abscessus* | 3A0122R |
| *Mycobacterium abscessus* | 3A0122S |
| *Mycobacterium abscessus* | 3A0731 |
| *Mycobacterium abscessus* | 3A0810R |
| *Mycobacterium abscessus* | 3A0930R |
| *Mycobacterium abscessus* | 3A0930S |
| *Mycobacterium abscessus* | 1S151_0930 |
| *Mycobacterium abscessus* | 1S152_0914 |
| *Mycobacterium abscessus* | 1S153_0915 |
| *Mycobacterium abscessus* | 1S154_0310 |
| *Mycobacterium abscessus* | 2B0107 |
| *Mycobacterium abscessus* | 2B0307 |
| *Mycobacterium abscessus* | 2B0626 |
| *Mycobacterium abscessus* | 2B0912R |
| *Mycobacterium abscessus* | 2B0912S |
| *Mycobacterium abscessus* | 2B1231 |
| *Mycobacterium abscessus* | 47J26 |
| *Mycobacterium abscessus* | 4S0116R |
| *Mycobacterium abscessus* | 4S0206 |
| *Mycobacterium abscessus* | 4S0303 |
| *Mycobacterium abscessus* | 4S0726RA |
| *Mycobacterium abscessus* | 4S0726RB |
| *Mycobacterium abscessus* | 5S0304 |
| *Mycobacterium abscessus* | 5S0421 |
| *Mycobacterium abscessus* | 5S0422 |
| *Mycobacterium abscessus* | 5S0708 |
| *Mycobacterium abscessus* | 5S0817 |
| *Mycobacterium abscessus* | 5S0921 |
| *Mycobacterium abscessus* | 5S1212 |
| *Mycobacterium abscessus* | 5S1215 |
| *Mycobacterium abscessus* | 6G0125R |
| *Mycobacterium abscessus* | 6G0125S |
| *Mycobacterium abscessus* | 6G0212 |
| *Mycobacterium abscessus* | 6G0728R |
| *Mycobacterium abscessus* | 6G0728S |
| *Mycobacterium abscessus* | 6G1108 |
| *Mycobacterium abscessus* | M115 |
| *Mycobacterium abscessus* | M139 |
| *Mycobacterium abscessus* | M148 |
| *Mycobacterium abscessus* | M152 |
| *Mycobacterium abscessus* | M154 |
| *Mycobacterium abscessus* | M156 |
| *Mycobacterium abscessus* | M159 |
| *Mycobacterium abscessus* | M172 |
| *Mycobacterium abscessus* | M18 |
| *Mycobacterium abscessus* | M24 |
| *Mycobacterium abscessus* | M93 |
| *Mycobacterium abscessus* | M94 |
| *Mycobacterium abscessus* | GO_06 |
| *Mycobacterium abscessus* | ATCC19977 |
| *Mycobacterium africanum* | GM041182 |
| *Mycobacterium avium* | 104 |
| *Mycobacterium avium* | ATCC25291 |
| *Mycobacterium avium* | paratuberculosis_K10 |
| *Mycobacterium avium* | paratuberculosis_S397 |
| *Mycobacterium avium* | paratuberculosis_S5 |
| *Mycobacterium canetti* | CIPT140010059 |
| *Mycobacterium canetti* | CIPT140060008 |
| *Mycobacterium canetti* | CIPT140070008 |
| *Mycobacterium canetti* | CIPT140070010 |
| *Mycobacterium canetti* | CIPT140070017 |
| *Mycobacterium chubuense* | NBB4 |
| *Mycobacterium colombiense* | CECT3035 |
| *Mycobacterium fortuitum* | DSM46621 |
| *Mycobacterium gilvum* | PYR_GCK |
| *Mycobacterium gilvum* | Spyr1 |
| *Mycobacterium hassicacum* | DSM44199 |
| *Mycobacterium indicus pranii* | MTCC9506 |
| *Mycobacterium intracellulare* | ATCC13950 |
| *Mycobacterium intracellulare* | Mi198_DNA |
| *Mycobacterium intracellulare* | MOTT02 |
| *Mycobacterium intracellulare* | MOTT64 |
| *Mycobacterium iranicum* | UM_TJL |
| *Mycobacterium kansasii* | ATCC12478 |
| *Mycobacterium leprae* | Br4923 |
| *Mycobacterium leprae* | TN |
| *Mycobacterium mageritense* | JR2009 |
| *Mycobacterium marinum* | M |
| *Mycobacterium parascrofulaceum* | ATCC_BAA_614 |
| *Mycobacterium phlei* | RIVM601174 |
| *Mycobacterium rhodesiae* | JS60 |
| *Mycobacterium rhodesiae* | NBB3 |
| *Mycobacterium smegmatis* | JS623 |
| *Mycobacterium smegmatis* | MC2_155_uid171958 |
| *Mycobacterium smegmatis* | MC2_155_uid57701 |
| *Mycobacterium thermoresistibile* | ATCC19527 |
| *Mycobacterium tuberculosis* | 210 |
| *Mycobacterium tuberculosis* | 94_M4241A |
| *Mycobacterium tuberculosis* | 98_R604_INH_RIF_EM |
| *Mycobacterium tuberculosis* | CCDC5079 |
| *Mycobacterium tuberculosis* | CCDC5180 |
| *Mycobacterium tuberculosis* | CCUG48898_uid180742 |
| *Mycobacterium tuberculosis* | CCUG48898_uid80701 |
| *Mycobacterium tuberculosis* | CDC1551A |
| *Mycobacterium tuberculosis* | CDC1551 |
| *Mycobacterium tuberculosis* | CPHL_A |
| *Mycobacterium tuberculosis* | CTRI_2 |
| *Mycobacterium tuberculosis* | EAS054 |
| *Mycobacterium tuberculosis* | F11 |
| *Mycobacterium tuberculosis* | GM1503 |
| *Mycobacterium tuberculosis* | H37Ra_Draft |
| *Mycobacterium tuberculosis* | H37Ra |
| *Mycobacterium tuberculosis* | H37Rv_uid170532 |
| *Mycobacterium tuberculosis* | H37Rv_uid57777 |
| *Mycobacterium tuberculosis* | Haarlem |
| *Mycobacterium tuberculosis* | K85 |
| *Mycobacterium tuberculosis* | KZN1435 |
| *Mycobacterium tuberculosis* | KZN4207_Draft |
| *Mycobacterium tuberculosis* | KZN4207 |
| *Mycobacterium tuberculosis* | KZN605 |
| *Mycobacterium tuberculosis* | KZN_R506 |
| *Mycobacterium tuberculosis* | KZN_V2475 |
| *Mycobacterium tuberculosis* | NCGM2209 |
| *Mycobacterium tuberculosis* | RGTB327 |
| *Mycobacterium tuberculosis* | RGTB423 |
| *Mycobacterium tuberculosis* | SUMu001 |
| *Mycobacterium tuberculosis* | SUMu002 |
| *Mycobacterium tuberculosis* | SUMu003 |
| *Mycobacterium tuberculosis* | SUMu004 |
| *Mycobacterium tuberculosis* | SUMu005 |
| *Mycobacterium tuberculosis* | SUMu006 |
| *Mycobacterium tuberculosis* | SUMu007 |
| *Mycobacterium tuberculosis* | SUMu008 |
| *Mycobacterium tuberculosis* | SUMu009 |
| *Mycobacterium tuberculosis* | SUMu010 |
| *Mycobacterium tuberculosis* | SUMu011 |
| *Mycobacterium tuberculosis* | SUMu012 |
| *Mycobacterium tuberculosis* | T17 |
| *Mycobacterium tuberculosis* | T46 |
| *Mycobacterium tuberculosis* | T85 |
| *Mycobacterium tuberculosis* | T92 |
| *Mycobacterium tuberculosis* | UT205 |
| *Mycobacterium tuberculosis* | W148 |
| *Mycobacterium tuberculosis* | 02_1987 |
| *Mycobacterium tusciae* | JS617 |
| *Mycobacterium ulcerans* | Agy99 |
| *Mycobacterium vaccae* | ATCC25954 |
| *Mycobacterium vanbaalenii* | PYR1 |
| *Mycobacterium xenopi* | RIVM700367 |
| *Mycobacterium*. sp | JLS |
| *Mycobacterium.* sp | KMS |
| *Mycobacterium.* sp | MCS |
| *Mycobacterium.* sp | MOTT36Y |
