# Supplementary material for: Whole-Genome Sequencing and Comparative Analysis of Mycobacterium brisbanense Reveals a Possible Soil Origin and Capability in Fertiliser Synthesis
Source: PLoS One. 2016 Mar 31;11(3):e0152682. doi: 10.1371/journal.pone.0152682 (PMC4816395; doi:10.1371/journal.pone.0152682)
Supplement: S4 Table — “/” means presence of the predicted virulence gene. “-” means absence of the predicted virulence gene. (DOCX) [file pone.0152682.s009.docx]

**S4 Table: Predicted virulence genes across the UM_WWY and 30 other mycobacterial genomes.** “/” means presence of the predicted virulence gene. “-” means absence of the predicted virulence gene.

1 – UM_WWY 2 – *M. abscessus*

3 – *M. africanum* 4 – *M. avium*

5 – *M. canetti* 6 – *M. chubuense*

7 – *M. colombiense* 8 – *M. fortuitum*

9 – *M. gilvum* 10 – *M. hassiacum*

11 – *M. indicus pranii* 12 – *M. intracellulare*

13 – *M. kansasii* 14 – *M. leprae*

15 – *M. mageritense* 16 – *M. marinum*

17 – *M. parascrofulaceum* 18 – *M. phlei*

19 – *M. rhodesiae* 20 – *M. smegmatis*

21 – *M. thermoresistibile* 22 – *M. tuberculosis*

23 – *M. tusciae*  24 – *M. ulcerans*

25 – *M. vaccae*  26 – *M. vanbaalenii*

27 – *M. xenopi*  28 – *M.* sp JLS

29 – M. sp KMS 30 – *M.* sp MCS

31 – *M. iranicum*

|  | **1** | **2** | **3** | **4** | **5** | **6** | **7** | **8** | **9** | **10** | **11** | **12** | **13** | **14** | **15** | **16** | **17** | **18** | **19** | **20** | **21** | **22** | **23** | **24** | **25** | **26** | **27** | **28** | **29** | **30** | **31** |
| --- | --- | --- | --- | --- | --- | --- | --- | --- | --- | --- | --- | --- | --- | --- | --- | --- | --- | --- | --- | --- | --- | --- | --- | --- | --- | --- | --- | --- | --- | --- | --- |
| ***glnA1*** | / | / | / | / | / | / | / | / | / | / | / | / | / | / | / | / | / | / | / | / | / | / | / | / | / | / | / | / | / | / | / |
| ***leuD*** | / | / | / | / | / | / | / | / | / | / | / | / | / | / | / | / | / | / | / | / | / | / | / | / | / | / | / | / | / | / | / |
| ***lysA*** | / | / | / | / | / | / | / | / | / | / | / | / | / | / | / | / | / | / | / | / | / | / | / | / | / | / | / | / | / | / | / |
| ***proC*** | / | / | / | / | / | / | / | / | / | / | / | / | / | / | / | / | / | / | / | / | / | / | / | / | / | / | / | / | / | / | / |
| ***purC*** | / | / | / | / | / | / | / | / | / | / | / | / | / | / | / | / | / | / | / | / | / | / | / | / | / | / | / | / | / | / | / |
| ***trpD*** | - | / | - | - | - | - | - | - | - | - | - | - | - | / | - | - | - | - | - | - | - | - | - | / | - | - | - | - | - | - |  |
| ***narX*** |  | - | / | - | - | - | - | - | - | - | - | - | - | - | - | - | - | - | - | - | - | / | - | - | - | - | - | - | - | - | - |
| ***narG*** | - | - | / | / | / | / | / | / | / | - | / | / | / | - | / | - | / | - | / | - | / | / | / | - | / | / | / | / | / | / | - |
| ***narH*** | - | - | / | / | / | / | / | / | / | - | / | / | / | - | / | - | / | / | / | - | / | / | / | - | / | / | / | / | / | / | - |
| ***narJ*** | - | - | / | / | / | / | / | / | / | - | / | / | / | - | / | - | / | / | / | - | / | / | / | - | / | / | / | / | / | / | - |
| ***narI*** | - | - | / | / | / | / | / | / | / | - | / | / | / | - | / | - | / | / | / | - | / | / | / | - | / | / | / | / | / | / | - |
| ***narK2*** | / | / | / | - | / | / | - | / | / | / | - | - | / | - | / | - | - | / | / | / | / | / | / | - | / | / | / | / | / | / | / |
| ***nuoG*** | / | / | / | / | / | / | / | / | / | - | / | / | / | - | / | / | - | / | / | / | / | / | / | / | / | / | / | / | / | / | / |
| ***erp*** | / | / | / | / | / | / | / | / | / | / | / | / | - | / | - | / | - | / | / | / | / | / | / | / | / | / | - | / | / | / | / |
| ***hbhA*** | / | / | / | / | / | / | / | / | / | / | / | / | / | / | / | / | / | / | / | / | / | / | / | / | / | / | / | / | / | / | / |
| ***mma4*** | / | - | / | / | / | / | / | / | / | / | / | / | / | / | / | / | / | / | / | / | / | / | / | / | / | / | / | / | / | / | / |
| ***cmaA2*** | / | / | / | / | / | / | / | / | / | / | / | / | / | / | / | / | / | / | / | / | / | / | / | / | / | / | / | / | / | / | / |
| ***fadD26*** | / | - | / | - | / | / | / | / | / | - | - | - | / | / | - | / | - | - | - | / | - | / | - | / | / | / | / | / | / | / | / |
| ***fadD28*** | / | / | / | / | / | / | / | / | / | / | / | / | / | / | / | / | / | - | - | / | - | / | / | / | / | / | / | / | / | / | / |
| ***ppsA*** | - | - | / | - | / | / | - | - | / | - | - | - | / | / | - | / | - | - | - | / | - | / | - | / | - | / | - | / | / | / | / |
| ***ppsB*** | - | - | / | - | / | / | - | - | / | - | - | - | / | / | - | / | - | - | - | / | - | / | - | / | / | / | - | / | / | / | / |
| ***ppsC*** | - | - | / | - | / | - | - | - | - | - | - | - | / | / | - | / | - | - | - | - | - | / | - | / | - | - | - | / | / | / | - |
| ***ppsD*** | - | - | / | - | / | / | - | - | / | - | - | - | / | / | - | / | - | - | - | / | - | / | - | / | / | / | - | - | - | - | / |
| ***ppsE*** | - | - | / | - | / | / | - | - | / | - | - | - | / | / | - | / | - | - | - | / | - | / | - | / | / | / | - | / | / | / | / |
| ***papA5*** | - | - | / | - | / | / | - | - | / | - | - | - | / | / | - | / | - | - | - | - | - | / | - | / | / | / | - | / | / | / | / |
| ***mas*** | / | / | / | / | / | - | / | / | - | / | / | / | / | / | / | / | / | / | - | - | / | / | / | / | - | - | / | / | / | / | - |
| ***mmpL7*** | - | - | / | - | / | - | - | - | - | - | - | - | / | / | - | / | - | - | - | - | - | / | - | / | - | - | - | - | - | - | - |
| ***ddrA*** | - | - | / | / | / | / | / | - | / | - | / | / | / | / | - | / | / | / | / | / | - | / | / | / | / | / | - | / | / | / | / |
| ***ddrB*** | - | - | / | / | / | / | / | - | / | - | / | / | / | - | - | / | / | - | / | / | - | / | / | / | / | / | - | / | / | / | / |
| ***drrC*** | - | - | / | / | / | / | / | - | / | - | / | / | / | / | - | / | / | - | / | - | - | / | / | / | / | / | - | / | / | / | / |
| ***tesA*** | - | - | - | - | - | - | - | - | - | - | - | - | - | - | - | - | - | - | - | - | - | - | - | - | - | - | - | - | - | - | - |
| ***lppx*** | - | - | - | - | - | - | - | - | - | - | - | - | - | - | - | - | - | - | - | - | - | - | - | - | - | - | - | - | - | - | - |
| ***pks15*** | - | - | - | - | - | - | - | - | - | - | - | - | - | - | - | - | - | - | - | - | - | - | - | - | - | - | - | - | - | - | - |
| ***pks1*** | - | - | - | - | - | - | - | - | - | - | - | - | - | - | - | - | - | - | - | - | - | - | - | - | - | - | - | - | - | - | - |
| ***pks15/1*** | - | - | - | - | - | - | - | - | - | - | - | - | - | - | - | - | - | - | - | - | - | - | - | - | - | - | - | - | - | - | - |
| ***fadD22*** | - | - | - | - | - | - | - | - | - | - | - | - | - | - | - | - | - | - | - | - | - | - | - | - | - | - | - | - | - | - | - |
| ***fadD29*** | - | - | - | - | - | - | - | - | - | - | - | - | - | - | - | - | - | - | - | - | - | - | - | - | - | - | - | - | - | - | - |
| ***pcaA*** | / | / | / | / | / | / | / | / | / | / | / | / | / | / | / | / | / | / | / | / | / | / | / | / | / | / | / | / | / | / | / |
| ***stf0*** | / | / | / | / | / | - | / | / | - | / | / | / | / | - | / | / | / | / | / | / | - | / | - | - | / | - | / | / | / | / | - |
| ***papA2*** | / | / | / | / | / | / | / | / | / | - | / | / | - | - | / | - | / | / | - | / | - | / | / | - | - | / | - | / | / | / | / |
| ***papA1*** | / | - | / | - | / | - | / | - | - | - | - | - | - | - | / | - | / | / | - | - | - | / | - | - | - | - | - | / | / | / | / |
| ***pks2*** | / | / | / | / | / | - | / | / | - | / | / | / | / | / | / | / | / | / | - | - | / | / | / | / | - | - | / | / | / | / | / |
| ***mmpL8*** | / | - | / | - | / | - | / | - | - | - | - | - | - | - | / | - | / | / | - | - | - | / | - | - | - | - | - | / | / | / | / |
| ***kasB*** | / | / | / | / | / | / | / | / | / | / | / | / | / | / | / | / | / | / | / | / | / | / | / | / | / | / | / | / | / | / | / |
| ***icl*** | / | / | / | / | / | / | / | / | / | / | / | / | / | - | / | / | / | / | / | / | / | / | / | / | / | / | / | / | / | / | / |
| ***lipF*** | - | - | / | / | / | / | / | / | / | / | / | / | / | / | / | / | / | / | / | / | / | / | / | / | / | / | / | / | / | / | / |
| ***sapM*** | - | - | / | / | / | / | / | - | - | - | / | / | / | - | - | / | / | - | - | / | - | / | - | / | - | - | / | - | - | - | - |
| ***panC*** | / | / | / | / | / | / | / | / | / | / | / | / | / | / | / | / | / | / | / | / | / | / | / | / | / | / | / | / | / | / | / |
| ***panD*** | / | / | / | / | / | / | / | / | / | / | / | / | / | / | / | / | / | / | / | / | / | / | / | / | - | / | / | / | / | / | - |
| ***plcA*** | - | - | / | - | / | - | - | - | - | - | - | - | / | - | - | / | - | - | - | - | - | / | - | / | - | - | - | - | - | - | - |
| ***plcB*** | - | - | / | - | / | - | - | - | - | - | - | - | / | - | - | / | - | - | - | - | - | / | - | / | - | - | - | - | - | - | - |
| ***plcC*** | - | - | / | - | / | - | - | - | - | - | - | - | / | - | - | / | - | - | - | - | - | / | - | / | - | - | - | - | - | - | - |
| ***plcD*** | - | - | / | - | / | - | - | - | - | - | - | - | / | - | - | / | - | - | - | - | - | / | - | / | - | - | - | - | - | - | - |
| ***mce1A*** | / | - | / | / | / | / | / | / | / | / | / | / | / | / | / | / | / | / | / | / | / | / | / | / | / | / | / | / | / | / | / |
| ***mce1B*** | / | - | / | / | / | / | / | / | / | / | / | / | / | / | / | / | / | / | / | / | / | / | / | / | / | / | / | / | / | / | / |
| ***mce1C*** | / | - | / | / | / | / | / | / | / | / | / | / | - | / | / | / | / | / | / | / | / | / | / | / | / | / | / | / | / | / | / |
| ***mce1D*** | / | - | / | / | / | / | / | / | / | / | / | / | / | / | / | / | / | / | / | / | / | / | / | / | / | / | / | / | / | / | / |
| ***mce1E*** | / | - | / | / | / | / | / | / | / | / | / | / | / | / | / | / | / | / | / | / | / | / | / | / | / | / | / | / | / | / | / |
| ***mce1F*** | / | - | / | / | / | / | / | / | / | / | / | / | / | / | / | / | / | / | / | / | / | / | / | / | / | / | / | / | / | / | / |
| ***mce2A*** | / | - | / | / | / | / | / | / | / | / | / | / | / | / | / | / | / | / | / | / | / | / | / | / | / | / | / | / | / | / | / |
| ***mce2B*** | / | - | / | / | / | / | / | / | / | / | / | / | / | / | / | / | / | / | / | / | / | / | / | / | / | / | / | / | / | / | / |
| ***mce2C*** | / | - | / | / | / | / | / | / | / | / | / | / | - | / | / | / | / | / | / | / | / | / | / | / | / | / | / | / | / | / | / |
| ***mce2D*** | / | - | / | / | / | / | / | / | / | / | / | / | / | / | / | / | / | / | / | / | / | / | / | / | / | / | / | / | / | / | / |
| ***mce2E*** | / | - | / | / | / | / | / | / | / | / | / | / | / | / | / | / | / | / | / | / | / | / | / | / | / | / | / | / | / | / | / |
| ***mce2F*** | / | - | / | / | / | / | / | / | / | / | / | / | / | / | / | / | / | / | / | / | / | / | / | / | / | / | / | / | / | / | / |
| ***mce3A*** | - | - | - | / | / | / | / | / | / | / | - | / | / | - | - | / | / | / | / | / | / | / | / | / | / | / | - | / | / | / | / |
| ***mce3B*** | - | - | - | / | / | / | / | / | / | / | - | / | / | - | - | / | / | / | / | / | / | / | / | / | / | / | - | / | / | / | / |
| ***mce3C*** | - | - | - | / | / | / | / | / | / | / | - | / | - | - | - | / | - | / | / | / | / | / | / | / | / | / | - | / | / | / | / |
| ***mce3D*** | - | - | - | / | / | / | / | / | / | / | - | / | / | - | - | / | / | / | / | / | / | / | / | / | / | / | - | / | / | / | / |
| ***mce3E*** | - | - | - | / | / | / | / | / | / | / | - | / | / | - | - | / | / | / | / | / | / | / | / | / | / | / | - | / | / | / | / |
| ***mce3F*** | - | - | - | / | / | / | / | / | / | / | - | / | / | - | - | / | / | / | / | / | / | / | / | / | / | / | - | / | / | / | / |
| ***mce4A*** | / | / | / | / | / | / | / | / | / | / | / | / | / | - | / | / | / | / | / | / | / | / | / | / | / | / | / | / | / | / | / |
| ***mce4B*** | / | / | / | / | / | / | / | / | / | / | / | / | / | - | / | / | / | / | / | / | / | / | / | / | / | / | / | / | / | / | / |
| ***mce4C*** | / | / | / | / | / | / | / | / | / | / | / | / | / | - | / | / | / | / | / | / | / | / | / | / | / | / | / | / | / | / | / |
| ***mce4D*** | / | / | / | / | / | / | / | / | / | / | / | / | / | - | / | / | / | / | / | / | / | / | / | / | / | / | / | / | / | / | / |
| ***mce4E*** | / | / | / | / | / | / | / | / | / | / | / | / | / | - | / | / | / | / | / | / | / | / | / | / | / | / | / | / | / | / | / |
| ***mce4F*** | / | / | / | / | / | / | / | / | / | / | / | / | / | - | - | / | / | / | / | / | / | / | / | / | / | / | / | / | / | / | / |
| ***mce5A*** | - | / | - | / | - | - | / | / | / | / | / | / | - | - | / | / | / | / | - | - | / | - | / | / | / | / | - | - | - | - | / |
| ***mce5B*** | - | / | - | / | - | - | / | / | / | / | / | / | - | - | / | / | / | / | - | - | / | - | / | / | / | / | - | - | - | - | / |
| ***mce5C*** | - | / | - | / | - | - | / | / | / | / | / | / | - | - | / | / | / | / | - | - | / | - | / | / | / | / | - | - | - | - | / |
| ***mce5D*** | - | / | - | / | - | - | / | / | / | / | / | / | - | - | / | / | / | / | - | - | / | - | / | / | / | / | - | - | - | - | / |
| ***mce5E*** | - | / | - | / | - | - | / | / | / | / | / | / | - | - | / | / | - | / | - | - | / | - | / | / | / | / | - | - | - | - | / |
| ***mce5F*** | - | / | - | / | - | - | / | / | / | / | / | / | - | - | / | / | / | / | - | - | / | - | / | / | / | / | - | - | - | - | / |
| ***mce6A*** | - | / | - | / | - | - | / | / | - | / | / | / | - | - | / | / | / | / | - | - | / | - | - | / | / | / | - | - | - | - | / |
| ***mce6B*** | - | / | - | / | - | - | / | / | - | - | / | / | - | - | / | / | - | - | - | - | - | - | / | / | / | / | - | - | - | - | / |
| ***mce6C*** | - | / | - | - | - | - | - | / | - | / | - | - | - | - | / | / | - | - | - | - | - | - | - | / | - | - | - | - | - | - | - |
| ***mce6D*** | - | / | - | / | - | - | / | / | / | / | / | / | - | - | / | / | / | / | - | - | / | - | / | / | / | / | - | - | - | - | / |
| ***mce6E*** | - | / | - | / | - | - | / | / | / | - | / | / | - | - | / | / | - | - | - | - | / | - | / | / | / | / | - | - | - | - | / |
| ***mce6F*** | - | / | - | / | - | - | / | / | / | - | / | / | - | - | / | / | / | / | - | - | / | - | / | / | / | / | - | - | - | - | / |
| ***mce7A*** | - | - | - | / | - | / | / | / | / | / | / | / | / | - | / | / | / | / | / | / | / | - | / | - | / | / | / | / | / | / | / |
| ***mce7B*** | - | - | - | / | - | / | / | / | / | / | / | / | / | - | / | / | / | / | / | / | / | - | / | - | / | / | / | / | / | / | / |
| ***mce7C*** | - | - | - | / | - | / | / | / | / | / | / | / | / | - | / | / | / | / | / | / | / | - | / | - | / | / | / | / | / | / | / |
| ***mce7D*** | - | - | - | / | - | / | / | / | / | / | / | / | / | - | / | / | / | / | / | / | / | - | / | - | / | / | / | / | / | / | / |
| ***mce7E*** | - | - | - | / | - | / | / | / | / | / | / | / | - | - | / | / | / | / | / | / | / | - | / | - | / | / | / | / | / | / | / |
| ***mce7F*** | - | - | - | / | - | / | / | / | / | / | / | / | / | - | / | / | / | / | / | / | / | - | / | - | / | / | / | / | / | / | / |
| ***mce8A*** | - | - | - | / | - | / | / | / | / | / | / | / | / | - | / | / | / | / | - | / | / | - | - | - | / | / | / | / | / | / | - |
| ***mce8B*** | - | - | - | / | - | / | / | / | / | / | / | / | / | - | / | / | / | / | / | / | / | - | / | - | / | / | / | / | / | / | / |
| ***mce8C*** | - | - | - | / | - | / | / | / | / | / | / | / | / | - | / | / | / | / | / | / | / | - | / | - | / | / | / | / | / | / | / |
| ***mce8D*** | - | - | - | / | - | / | / | / | / | / | / | / | / | - | / | / | / | / | / | / | / | - | / | - | / | / | / | / | / | / | / |
| ***mce8E*** | - | - | - | / | - | / | / | / | / | / | / | / | - | - | / | / | / | / | / | / | / | - | / | - | / | / | / | / | / | / | / |
| ***mce8F*** | - | - | - | / | - | / | / | / | / | / | / | / | / | - | / | / | / | / | / | / | / | - | / | - | / | / | / | / | / | / | / |
| ***mce9A*** | - | / | - | / | - | - | / | / | / | / | / | / | - | - | / | / | / | / | - | - | / | - | / | / | / | / | - | - | - | - | / |
| ***mce9B*** | - | / | - | / | - | - | / | / | / | / | / | / | - | - | / | / | - | / | - | - | / | - | / | / | / | / | - | - | - | - | / |
| ***mce9C*** | - | / | - | / | - | - | / | / | / | / | / | / | - | - | / | / | / | / | - | - | / | - | / | / | / | / | - | - | - | - | - |
| ***mce9D*** | - | / | - | / | - | - | / | / | / | / | / | / | - | - | / | / | / | / | - | - | / | - | / | / | / | / | - | - | - | - | / |
| ***mce9E*** | - | / | - | / | - | - | / | / | / | - | / | / | - | - | / | / | - | / | - | - | / | - | / | / | / | / | - | - | - | - | / |
| ***mce9F*** | - | / | - | / | - | - | / | / | / | - | / | / | - | - | / | / | / | / | - | - | / | - | / | / | / | / | - | - | - | - | / |
| ***irtA*** | / | / | / | / | / | - | / | / | - | - | / | / | - | - | / | / | / | - | / | - | / | / | / | / | - | - | - | - | - | - | - |
| ***irtB*** | / | / | / | / | / | - | / | / | - | - | / | / | - | - | / | / | / | - | / | - | / | / | / | / | - | - | - | - | - | - | - |
| ***fxuC*** | - | - | - | - | - | - | - | - | - | - | - | - | - | - | - | - | - | - | - | - | - | - | - | - | - | - | - | - | - | - | - |
| ***fxuA*** | - | - | - | - | - | - | - | - | - | - | - | - | - | - | - | - | - | - | - | - | - | - | - | - | - | - | - | - | - | - | - |
| ***fxuB*** | - | - | - | - | - | - | - | - | - | - | - | - | - | - | - | - | - | - | - | - | - | - | - | - | - | - | - | - | - | - | - |
| ***fxbA*** | - | - | - | - | - | - | - | - | - | - | - | - | - | - | - | - | - | - | - | - | - | - | - | - | - | - | - | - | - | - | - |
| ***exiT*** | - | - | - | - | - | - | - | - | - | - | - | - | - | - | - | - | - | - | - | - | - | - | - | - | - | - | - | - | - | - | - |
| ***fxbBC*** | - | - | - | - | - | - | - | - | - | - | - | - | - | - | - | - | - | - | - | - | - | - | - | - | - | - | - | - | - | - | - |
| ***fxuD*** | - | - | - | - | - | - | - | - | - | - | - | - | - | - | - | - | - | - | - | - | - | - | - | - | - | - | - | - | - | - | - |
| ***ideR*** | / | / | / | / | / | / | / | / | / | / | / | / | / | / | / | / | / | / | / | / | / | / | / | / | / | / | / | / | / | / | / |
| ***mgtC*** | / | / | / | / | / | - | / | - | - | - | / | / | / | - | - | / | / | - | - | - | - | / | - | / | - | - | / | / | / | / | - |
| ***mbtH*** | / | / | / | / | / | / | / | / | / | / | / | / | / | - | / | / | / | / | / | / | / | / | / | / | / | / | / | / | / | / | / |
| ***mbtG*** | - | / | / | / | / | - | / | / | / | / | / | / | / | - | - | / | / | / | / | - | / | / | / | / | / | / | / | / | / | / | / |
| ***mbtF*** | / | / | / | / | / | - | / | / | / | / | / | / | / | - | / | / | / | / | / | - | / | / | / | / | / | / | - | / | / | / | / |
| ***mbtE*** | / | / | / | / | / | - | / | / | / | / | / | / | / | - | / | / | / | / | / | - | / | / | / | / | / | / | / | / | / | / | / |
| ***mbtD*** | / | / | / | / | / | - | / | / | / | - | / | / | / | - | / | / | / | - | - | - | / | / | - | / | / | / | - | / | / | / | / |
| ***mbtC*** | / | / | / | / | / | - | / | / | / | / | / | / | / | - | - | / | / | / | / | - | / | / | / | / | / | / | / | / | / | / | / |
| ***mbtB*** | / | / | / | / | / | - | / | / | / | / | / | / | / | - | / | / | / | / | / | - | / | / | / | / | / | / | / | / | / | / | / |
| ***mbtA*** | / | / | / | / | / | - | / | / | / | / | / | / | / | - | / | / | / | / | / | - | / | / | / | / | / | / | / | / | / | / | / |
| ***mbtJ*** | / | / | / | / | / | / | / | / | - | - | / | / | / | - | / | / | - | - | / | / | - | / | - | / | - | - | / | - | - | - | / |
| ***mbtI*** | / | / | / | / | / | - | / | / | - | - | / | / | / | - | / | / | / | - | - | - | - | / | - | / | - | - | / | - | - | - | - |
| ***fadD33*** | / | - | / | / | / | - | - | / | / | / | / | / | - | - | / | - | - | / | - | - | / | / | / | - | / | / | - | / | / | / | / |
| ***fadE14*** | / | - | / | / | / | - | / | / | - | / | / | / | - | - | - | - | - | / | - | - | / | / | / | - | / | / | - | / | / | / | / |
| ***relA*** | / | / | / | / | / | / | / | / | / | / | / | / | / | / | / | / | / | / | / | / | / | / | / | / | / | / | / | / | / | / | / |
| ***devR/dosR*** | / | / | / | / | / | / | / | / | / | / | - | - | / | - | / | / | / | / | / | - | / | / | / | / | / | / | / | / | / | / | / |
| ***devS*** | / | / | / | / | / | / | / | / | / | / | - | - | / | - | / | / | / | / | / | - | / | / | / | / | / | / | / | / | / | / | / |
| ***mprA*** | / | / | / | / | / | / | / | / | / | / | / | / | / | / | / | / | / | / | / | / | / | / | / | / | / | / | / | / | / | / | / |
| ***mprB*** | / | / | / | / | / | / | / | / | / | / | / | / | / | / | / | / | / | / | / | / | / | / | / | / | / | / | / | / | / | / | / |
| ***phoP*** | / | / | / | / | / | / | / | / | / | - | / | / | / | - | / | / | / | / | / | / | - | / | / | / | / | / | / | / | / | / | / |
| ***phoR*** | / | / | / | / | / | / | / | / | / | - | / | / | / | - | / | / | / | / | / | / | - | / | / | / | / | / | / | / | / | / | / |
| ***prrA*** | / | / | / | / | / | / | / | / | / | / | / | / | / | / | / | / | / | / | / | / | / | / | / | / | / | / | / | / | / | / | / |
| ***prrB*** | / | / | / | / | / | / | / | / | / | / | / | / | / | / | / | / | / | / | / | / | / | / | / | / | / | / | / | / | / | / | / |
| ***sigA/rpoV*** | / | / | / | / | / | / | / | / | / | / | / | / | / | / | - | / | / | / | / | / | / | / | / | / | / | / | / | / | / | / | / |
| ***sigE*** | / | / | / | / | / | / | / | / | / | / | / | / | / | / | / | / | / | / | / | / | / | / | / | / | / | / | / | / | / | / | / |
| ***sigF*** | / | / | / | / | / | / | / | / | / | / | / | / | / | - | / | / | / | / | / | / | / | / | / | / | / | / | / | / | / | / | / |
| ***sigH*** | / | / | / | / | / | / | / | / | / | / | / | / | / | - | / | / | / | / | / | / | / | / | / | / | / | / | / | / | / | / | / |
| ***sigM*** | / | / | / | / | / | / | / | / | / | / | / | / | / | - | / | / | / | / | / | / | / | / | / | / | / | / | / | / | / | / | / |
| ***whiB3*** | / | / | / | / | - | / | / | / | / | / | / | / | / | / | - | / | / | / | / | / | / | - | - | / | / | / | / | - | - | - | - |
| ***lpqH*** | / | / | / | / | / | / | / | / | / | / | / | / | / | / | / | / | / | / | / | / | / | / | / | / | / | / | - | / | / | / | / |
| ***hspX*** | / | / | / | - | / | / | / | - | - | / | / | / | / | - | - | / | / | / | / | - | / | / | - | - | / | / | / | / | / | / | / |
| ***fbpA*** | / | / | / | / | / | / | / | / | / | / | / | / | / | / | / | / | / | / | / | / | / | / | / | / | / | / | / | / | / | / | / |
| ***fbpB*** | / | / | / | / | / | / | / | / | / | / | / | / | / | / | / | / | / | / | / | / | / | / | / | / | / | / | / | / | / | / | / |
| ***fbpC*** | / | / | / | / | / | / | / | / | / | / | / | / | / | / | / | / | / | / | / | / | / | / | / | / | / | / | / | / | / | / | / |
| ***eis*** | - | / | / | - | / | - | - | / | / | - | - | - | / | - | / | / | - | / | - | - | - | / | - | - | / | / | - | / | / | / | - |
| ***pknG*** | / | / | / | / | / | / | / | / | / | / | / | / | / | / | / | / | / | / | / | / | / | / | / | / | / | / | / | / | / | / | / |
| ***secA2*** | / | / | / | / | / | / | / | / | / | / | / | / | / | / | / | / | / | / | / | / | / | / | / | / | / | / | / | / | / | / | / |
| ***esxA*** | / | - | / | - | / | - | - | / | / | / | - | - | / | / | / | / | - | / | / | - | / | / | / | - | / | / | - | / | / | / | / |
| ***esxB*** | / | - | / | - | / | - | - | / | / | / | - | - | / | - | / | / | - | / | / | - | / | / | / | - | - | / | - | / | / | / | - |
| ***eccA1*** | / | - | / | - | / | - | - | / | / | / | - | - | / | / | / | / | - | / | / | - | / | / | / | - | / | / | - | / | / | / | / |
| ***eccB1*** | / | - | / | - | / | - | - | / | / | / | - | - | / | / | - | / | - | / | / | - | / | / | / | - | / | / | - | / | / | / | / |
| ***eccCa1*** | / | - | / | - | / | - | - | / | / | / | - | - | / | / | / | / | - | / | / | - | / | / | / | - | / | / | - | / | / | / | / |
| ***eccCb1*** | / | - | / | - | / | - | - | / | / | / | - | - | / | / | / | / | - | / | / | - | / | / | / | - | / | / | - | / | / | / | / |
| ***PE35*** | / | - | / | - | / | - | - | / | / | / | - | - | / | - | / | / | - | / | / | - | / | / | / | - | / | / | - | / | / | / | / |
| ***eccD1*** | / | - | / | - | / | / | - | / | / | / | - | - | / | / | / | / | - | / | / | - | / | / | / | / | / | / | - | / | / | / | / |
| ***espK*** | - | - | - | - | - | - | - | - | - | - | - | - | / | - | - | / | - | - | - | - | - | - | - | - | - | - | - | - | - | - | - |
| ***eccE1*** | / | - | / | - | / | / | - | / | / | / | - | - | / | / | / | / | - | / | / | - | / | / | / | - | / | / | - | / | / | / | / |
| ***mycP1*** | / | - | / | - | / | / | - | / | / | / | - | - | / | / | / | / | - | / | / | - | / | / | / | - | / | / | - | / | / | / | / |
| ***espD*** | - | - | / | - | / | - | - | - | - | - | - | - | - | / | - | / | - | - | - | - | - | / | - | - | - | - | - | - | - | - | - |
| ***espC*** | - | - | / | - | / | - | - | - | - | - | - | - | / | / | - | / | - | - | - | - | - | / | - | - | - | - | - | - | - | - | - |
| ***espA*** | - | - | / | - | / | - | - | - | - | - | - | - | / | / | - | / | - | - | - | - | - | / | - | - | - | - | - | - | - | - | - |
| ***espB*** | - | - | / | - | / | - | - | - | / | - | - | - | / | - | - | / | - | - | - | - | - | / | - | - | / | / | - | / | / | / | / |
| ***PPE68*** | / | - | / | - | / | - | - | / | / | / | - | - | / | / | / | / | - | / | / | - | / | / | / | - | / | / | - | / | / | / | / |
| ***espI*** | / | / | / | / | / | / | / | / | / | / | / | / | / | / | / | / | / | / | / | / | / | / | / | / | / | / | / | / | / | / | / |
| ***espJ*** | - | - | / | - | / | - | - | - | / | - | - | - | / | - | - | / | - | - | - | - | - | / | - | / | / | / | - | - | - | - | - |
| ***espL*** | / | - | / | - | / | / | - | / | / | / | - | - | / | / | / | / | - | / | / | - | / | / | / | / | / | / | - | / | / | / | / |
| ***espR*** | / | / | / | / | / | / | / | / | / | / | / | / | / | / | / | / | / | / | / | / | / | / | / | / | / | / | / | / | / | / | / |
| ***eccA2*** | - | - | / | / | / | - | / | - | - | - | / | / | / | - | - | - | / | - | - | - | - | / | - | - | - | - | / | - | - | - | - |
| ***eccE2*** | - | - | / | / | / | - | / | - | - | - | / | / | / | - | - | - | / | - | - | - | - | / | - | - | - | - | - | - | - | - | - |
| ***mycP2*** | / | - | / | / | / | - | / | - | - | - | / | / | / | - | - | - | - | - | - | - | - | / | - | - | - | - | / | - | - | - | - |
| ***eccD2*** | - | - | / | / | / | - | / | - | - | - | / | / | / | - | - | - | / | - | - | - | - | / | - | - | - | - | - | - | - | - | - |
| ***espG2*** | - | - | / | / | / | - | / | - | - | - | / | / | / | - | - | - | / | - | - | - | - | / | - | - | - | - | - | - | - | - | - |
| ***esxC*** | - | - | - | / | / | - | / | - | - | - | / | / | / | - | - | - | / | - | - | - | - | / | - | - | - | - | - | - | - | - | - |
| ***esxD*** | - | - | / | / | / | - | / | - | - | - | / | / | / | - | - | - | / | - | - | - | - | / | - | - | - | - | - | - | - | - | - |
| ***PPE69*** | - | - | / | / | / | - | / | - | - | - | / | / | / | - | - | - | / | - | - | - | - | / | - | - | - | - | - | - | - | - | - |
| ***PE36*** | - | - | / | / | / | - | / | - | - | - | / | / | / | - | - | - | / | - | - | - | - | / | - | - | - | - | - | - | - | - | - |
| ***eccC2*** | - | - | / | / | / | - | / | - | - | - | / | / | / | - | - | - | / | - | - | - | - | / | - | - | - | - | / | - | - | - | - |
| ***eccB2*** | - | - | / | / | / | - | / | - | - | - | / | / | / | - | - | - | / | - | - | - | - | / | - | - | - | - | / | - | - | - | - |
| ***eccA3*** | / | / | / | / | / | - | / | / | / | / | / | / | / | / | / | / | / | / | / | - | / | / | / | / | / | / | / | / | / | / | / |
| ***eccB3*** | / | / | / | / | / | - | / | / | / | / | / | / | / | / | / | / | / | / | / | - | / | / | / | / | / | / | / | / | / | / | / |
| ***eccC3*** | / | / | / | / | / | - | / | / | / | / | / | / | / | / | - | / | / | / | / | - | / | / | / | / | / | / | / | / | / | / | / |
| ***PE5*** | / | / | / | / | / | - | / | / | / | / | / | / | / | / | / | / | / | / | / | - | / | / | / | / | / | / | / | / | / | / | / |
| ***PPE4*** | / | / | / | / | / | - | / | - | / | - | / | / | - | - | - | / | - | / | - | - | - | / | - | / | / | / | - | / | / | / | / |
| ***esxH*** | / | / | / | / | / | - | / | / | / | / | / | / | / | / | / | / | / | / | / | - | / | / | / | / | / | / | / | / | / | / | / |
| ***espG3*** | / | / | / | / | / | - | / | / | / | / | / | / | / | / | / | / | / | / | / | - | / | / | / | / | / | / | / | / | / | / | / |
| ***eccD3*** | / | / | / | / | / | - | / | / | / | / | / | / | / | / | / | / | - | / | / | - | / | / | / | / | / | / | / | / | / | / | / |
| ***mycP3*** | / | / | / | / | / | - | / | / | / | / | / | / | / | / | / | / | / | / | / | - | / | / | / | / | / | / | / | / | / | / | / |
| ***eccE3*** | / | / | / | / | / | - | / | / | / | - | / | / | / | / | / | / | / | - | - | - | - | / | - | / | / | / | / | / | / | / | / |
| ***esxG*** | / | / | / | / | / | - | / | / | / | / | / | / | / | / | / | / | / | / | / | - | / | / | / | / | / | / | / | / | / | / | / |
| ***esxT*** | / | - | / | / | / | / | / | / | / | / | / | / | / | - | / | / | / | / | / | - | / | / | / | / | / | / | / | / | / | / | / |
| ***esxU*** | / | - | / | / | / | / | / | / | / | / | / | / | / | - | / | / | / | / | / | - | / | / | / | / | / | / | / | / | / | / | / |
| ***eccC4*** | / | / | / | / | / | / | / | / | / | - | / | / | / | - | / | / | / | - | - | - | - | / | - | / | / | / | / | / | / | / | / |
| ***cccD4*** | - | / | / | / | / | - | - | - | - | - | - | - | - | - | - | / | - | - | - | - | - | / | - | / | - | / | - | / | / | / | - |
| ***mycP4*** | / | / | / | / | / | / | / | / | / | / | / | / | / | - | / | - | - | / | / | - | / | / | / | - | / | / | / | / | / | / | - |
| ***eccB4*** | / | / | / | / | / | / | / | - | / | - | / | / | / | - | - | / | / | - | - | - | - | / | - | / | / | / | - | / | / | / | / |
| ***eccA5*** | - | - | / | / | / | - | / | - | - | - | / | / | / | / | - | / | / | - | - | - | - | / | - | / | - | - | / | - | - | - | - |
| ***eccE5*** | - | - | / | / | / | - | / | - | - | - | / | / | / | / | - | / | / | - | - | - | - | / | - | / | - | - | - | - | - | - | - |
| ***mycP5*** | / | - | / | / | / | - | / | - | - | - | / | / | / | / | - | / | / | - | - | - | - | / | - | / | - | - | / | - | - | - | - |
| ***eccD5*** | - | - | / | / | / | - | / | - | - | - | / | / | / | / | - | / | / | - | - | - | - | / | - | / | - | - | / | - | - | - | - |
| ***esxN*** | / | - | / | / | / | - | / | - | - | - | / | / | / | / | - | / | / | - | - | - | - | / | - | / | - | - | / | - | - | - | - |
| ***esxM*** | / | - | / | / | / | - | / | - | - | - | / | / | / | / | - | / | / | - | - | - | - | / | - | / | - | - | / | - | - | - | - |
| ***eccCb5*** | / | - | / | / | / | - | / | - | - | - | / | / | / | / | - | / | / | - | - | - | - | / | - | / | - | - | / | - | - | - | - |
| ***eccCa5*** | / | - | / | / | / | - | / | - | - | - | / | / | / | / | - | / | / | - | - | - | - | / | - | / | - | - | / | - | - | - | - |
| ***eccB5*** | / | - | / | / | / | - | / | - | - | - | / | / | / | / | - | / | / | - | - | - | - | / | - | / | - | - | / | - | - | - | - |
| ***PPE41*** | - | - | / | - | / | - | - | - | - | - | - | - | - | - | - | - | - | - | - | - | - | / | - | - | - | - | - | - | - | - | - |
| ***PPE25*** | - | - | / | / | / | - | / | - | - | - | / | / | / | - | - | / | / | - | - | - | - | / | - | / | - | - | - | - | - | - | - |
| ***PE18*** | / | - | / | / | / | - | / | - | - | - | / | / | / | - | - | / | / | - | - | - | - | / | - | / | - | - | / | - | - | - | - |
| ***PPE26*** | - | - | / | / | / | - | / | - | - | - | / | / | / | - | - | / | / | - | - | - | - | / | - | / | - | - | - | - | - | - | - |
| ***PPE27*** | - | - | / | - | / | - | - | - | - | - | - | - | / | - | - | - | - | - | - | - | - | / | - | - | - | - | - | - | - | - | - |
| ***PE19*** | / | - | / | / | / | - | / | - | - | - | / | / | / | - | - | / | / | - | - | - | - | / | - | / | - | - | / | - | - | - | - |
| ***ahpC*** | / | / | / | / | / | - | / | - | - | - | / | / | / | / | - | / | / | - | - | - | / | / | - | / | - | - | / | - | - | - | - |
| ***katG*** | / | / | / | / | / | / | / | / | / | / | / | / | / | - | / | / | / | / | / | / | / | / | / | / | / | / | / | / | / | / | / |
| ***sodC*** | / | / | / | / | / | / | / | / | / | / | / | / | / | / | / | / | / | / | / | / | / | / | / | / | / | / | / | / | / | / | / |
| ***sodA*** | / | / | / | / | / | - | / | / | - | / | / | / | / | / | / | / | / | - | - | - | / | / | - | / | - | - | / | / | / | / | - |
| ***mlsA1*** | - | - | - | - | - | - | - | - | - | - | - | - | - | - | - | - | - | - | - | - | - | - | - | - | - | - | - | - | - | - | - |
| ***mlsA2*** | - | - | - | - | - | - | - | - | - | - | - | - | - | - | - | - | - | - | - | - | - | - | - | - | - | - | - | - | - | - | - |
| ***mlsB*** | - | - | - | - | - | - | - | - | - | - | - | - | - | - | - | - | - | - | - | - | - | - | - | - | - | - | - | - | - | - | - |
